# Analogue modulation of back-propagating action potentials enables dendritic hybrid signalling

**DOI:** 10.1038/ncomms13033

**Published:** 2016-10-05

**Authors:** János Brunner, János Szabadics

**Affiliations:** 1 Institute of Experimental Medicine, Hungarian Academy of Sciences, 43 Szigony Street, Budapest 1083, Hungary

## Abstract

We report that back-propagating action potentials (bAPs) are not simply digital feedback signals in dendrites but also carry analogue information about the overall state of neurons. Analogue information about the somatic membrane potential within a physiological range (from −78 to −64 mV) is retained by bAPs of dentate gyrus granule cells as different repolarization speeds in proximal dendrites and as different peak amplitudes in distal regions. These location-dependent waveform changes are reflected by local calcium influx, leading to proximal enhancement and distal attenuation during somatic hyperpolarization. The functional link between these retention and readout mechanisms of the analogue content of bAPs critically depends on high-voltage-activated, inactivating calcium channels. The hybrid bAP and calcium mechanisms report the phase of physiological somatic voltage fluctuations and modulate long-term synaptic plasticity in distal dendrites. Thus, bAPs are hybrid signals that relay somatic analogue information, which is detected by the dendrites in a location-dependent manner.

## Abstract (web summary)

‘Analogue' modulation by somatic membrane potentials can modify ‘digital' axonal action potentials. Here, the authors show that analogue modulation can occur in back-propagating dendritic action potentials and calcium signals, leading to signal enhancement or attenuation in a location-dependent manner.

## Graphical abstract

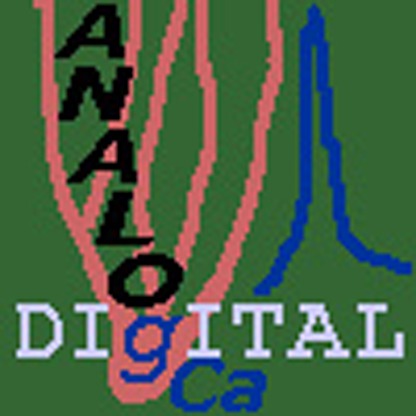

Action potentials (APs), the digital signals of neurons^1^, provide essential functions by converting incoming inputs to neuronal outputs^2^ along the dendro–somato–axonal axis. In addition to this straightforward direction, back-propagating action potentials (bAPs) carry a digital feedback to the synaptic input zone regarding the output activity of the neuron^3^ and participate in the integration, modulation and maintenance of synaptic inputs^4^. In contrast to APs, the somatic membrane potential is an analogue signal that dynamically reflects the overall input activity. However, in addition to the conventional analogue–digital–analogue conversion, recent studies in mammalian central neurons have demonstrated that analogue signals directly modulate the function of axonal APs, which enables them to act as hybrid signals^5^. Specifically, the axonal membrane potential modulates the efficacy of individual APs in evoking postsynaptic responses in an analogue manner^6^, ^7^, ^8^, ^9^, ^10^, ^11^, ^12^. Thus, the primary digital information (that is, presynaptic activity) is preserved, but the analogue content (that is, membrane potential) modifies the weight of the hybrid signal allowing for more information to be contained in single APs compared with conventional digital signalling^5^, ^13^. However, it is not known whether bAPs in dendrites are also capable of hybrid signalling.

At dendrites, local voltage changes modulate the properties^14^, ^15^, ^16^, ^17^, ^18^ and impact^19^, ^20^, ^21^ of bAPs, for example, by affecting the relief of Mg^2+^ block from *N*-methyl-D-aspartate (NMDA) receptors, which enables the spatially precise long-term synaptic plasticity^22^, ^23^, ^24^. However, these voltage changes do not spread into the entire dendritic tree because of the restricted propagation along the long dendritic cables. In contrast, the actual somatic voltage, which reflects the summed activity of inputs onto the complete dendritic tree, globally contributes to the dendritic voltage because of the physical and electrotonic dominance of the somatic membrane over the dendrites. Such asymmetric propagation potentially enables general somato-dendritic hybrid signalling and it is pronounced in dentate gyrus granule cells (GCs). Specifically, the short and proximally emerging individual branches with little active conductance^25^, ^26^, ^27^ make the dendritic tree of GCs ideal for monitoring the general activity through the soma. Importantly, somatic membrane potential comprises different information than local dendritic voltage. Although the somatic membrane potential is determined mostly by the locally larger dendritic excitatory postsynaptic potentials (EPSPs), it is the general reflection of the overall activity and state of GCs because it integrates these EPSPs and other types of inputs from every dendritic branch. Thus, it remained unanswered whether this general analogue content can be added to dendritic digital signalling as additional information.

Dendrites must fulfil three essential criteria for hybrid signalling. First, analogue information about the somatic membrane potential must be retained by the dendritic bAPs. Second, the analogue content cannot override the digital timing information of the bAPs. Third, dendritic mechanisms must reflect the analogue content of the hybrid bAP signals. Here we examine the possibility of dendritic hybrid information transfer in rat GCs and argue that their dendritic bAPs meet the criteria for hybrid signalling, and that this phenomenon endows the soma with a general activity-driven control over fundamental dendritic functions. First, the kinetics and amplitudes of the bAPs are influenced by the physiological somatic membrane potential. Second, these analogue state-dependent changes of the bAP waveforms are reflected by location-dependent changes in local dendritic calcium signals and long-term modulation of synapses. Furthermore, our results suggest that the analogue modulation of bAPs in the dendrites is mediated by multiple mechanisms that are coordinated in a location-dependent manner.

## Results

### Dual effects of somatic potential on dendritic bAP waveforms

**Figure 1:**
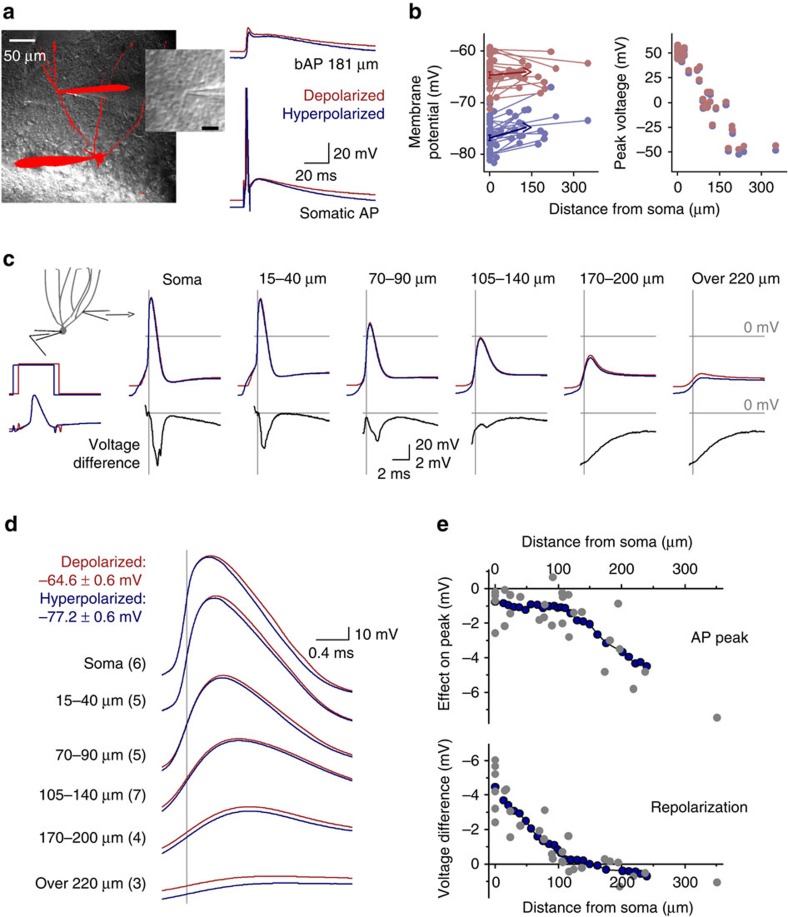
Location-dependent effects of somatic membrane potential on bAP waveforms. (**a**) Superimposed IR-DIC and confocal *z* stack images of a GC, which was simultaneously recorded at a somatic and a dendritic site. Spikes were evoked with short current injections at the soma and the membrane potential was also set at this location with steady-state current injections to slightly depolarized (red, −64.6±0.6 mV, *n*=29) or hyperpolarized (blue, −77.2±0.6 mV) potentials. The second pipette was used for monitoring the undisturbed voltage either at a dendrite or at the soma. (**b**) Propagation of somatically set membrane potential into dendrites (left panel). Connected symbols indicate individual experiments. Arrows show the average vector of the membrane potential difference. The right panel shows the distance dependence of the absolute bAP peak voltage (see also ref. 26). (**c**) Average bAP waveforms at various monitored locations. Representative somatic traces are shown on the left. Spikes were aligned to their maximum rate of rise (vertical grey lines). Black traces below show the voltage difference between the bAPs evoked from hyperpolarized and depolarized potentials. (**d**) The same average bAPs as in **c** on different timescale. Numbers show the included experiment numbers. (**e**) Effects of hyperpolarization on the peak voltage and relative repolarization of bAPs. Grey symbols indicate individual experiments, whose running average is shown in blue (calculated from 9 neighboring data points). To quantify the differences during the repolarization relative to the loss of the peak voltage, the voltage difference between the two bAPs at 0.2–1.2 ms after the peak was compared with the voltage difference at the peak.

To determine whether bAPs retain analogue information about the somatic membrane potential, we simultaneously recorded single bAPs from the dendrites and soma of GCs. During these experiments, the thin dendrites were patched first under infrared differential interference contrast (IR-DIC) optics. The pipettes contained Alexa Fluor 594 dye for the visualization and patching the parent soma (Fig. 1c). Only the somatic pipette was used for current injection to control physiologically plausible depolarized (−64.6±0.6 mV) or hyperpolarized (−77.2±0.6 mV) membrane potentials^28^, ^29^, ^30^ and to evoke single action potentials. Because of the reliable voltage monitoring (Supplementary Fig. 1) and intermingled sampling of the two conditions these high series resistance recordings allowed the direct comparison of bAP shapes. First, we confirmed previous observations that somatic steady-state voltage spreads effectively into the dendrites of GCs^26^, ^27^, and that the peak amplitudes of the bAPs decrease toward the tips of the dendrites (Fig. 1b; Supplementary Fig. 2). To directly compare bAP waveforms at two physiologically relevant membrane potentials, the elicited bAP pairs were aligned to the maximum rate of rise (Fig. 1c). The apparent threshold, rising phase and absolute peak voltage of the bAPs in the proximal locations were unchanged regardless of the preceding membrane potential, which further confirms the reliability of the recording conditions. However, there was a transient voltage difference between the depolarized and hyperpolarized bAPs during their repolarization phase (Fig. 1c–e). The proximal bAPs repolarized faster when they were evoked from hyperpolarized membrane potentials. In contrast to proximal dendrites, the active components at distal locations were not sufficient to compensate for the initial membrane potential difference; thus, the bAPs from hyperpolarized membrane potentials reached lower peak voltages than the bAPs from depolarized membrane potentials (Fig. 1c–e). Furthermore, hyperpolarization did not have differential effects on the repolarization of the distal bAPs relative to their peaks. Altogether, the dual recordings indicate that analogue information regarding the somatic membrane potential in GCs is location-dependently retained as differences in the dendritic bAP waveform.

### Bidirectional effects of somatic potential on calcium signals

**Figure 2:**
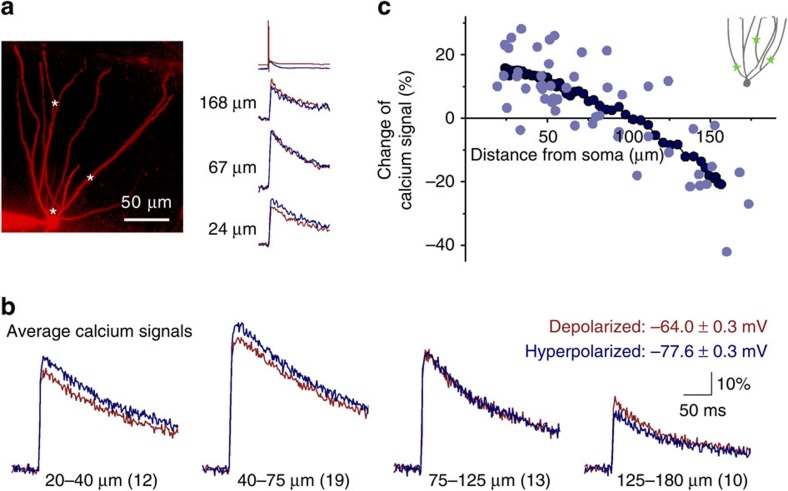
Location-dependent bidirectional effects of somatic membrane potential on bAP-evoked dendritic calcium signals. (**a**) Representative experiment showing the three imaged locations (24, 67 and 168 μm) of a GC filled with calcium-sensitive Fluo-5F (183 μM) and Alexa Fluor 594 (15 μM) dyes loaded by somatic patch pipette, which was also used for setting the somatic membrane potential. Imaging traces (Δ*F*/*F*_0_) from the Fluo-5F channel were alternately recorded at the two membrane potentials (−65.1 and −77.6 mV). Three-dimensional *z* stack Alexa Fluor 594 imaging was performed after the end of the calcium measurement and short lower intensity imaging in the red channel was used to set and maintain the line scan positions. (**b**) Average calcium imaging trace pairs (Δ*F*/*F*_0_) along the dendrites at two somatic membrane potentials (−64.0±0.3 and −77.6±0.3 mV). Note that perisomatic imaging (<20 μm) was avoided to prevent photo-damage and that in the most distal regions bAPs do not evoke detectable calcium signals in GCs (Supplementary Fig. 3; see also ref. 26). (**c**) Light blue symbols mark the effect of hyperpolarization on local calcium signals along the dendritic distance in individual experiments (*n*=54 spots from *n*=25 cells) and dark blue symbols show the running average (13 individual point were used for each average data points).

Do these bAP waveform changes have any functional consequence in the dendrites? One of the most important functions of bAPs is to evoke local calcium influx; the calcium ions that enter are crucial in various mechanisms, including synaptic plasticity, local excitability and cellular metabolism^4^. Therefore, to determine whether and how somatic membrane potential is reflected by bAP-evoked local calcium influx, we used conventional confocal imaging (Supplementary Fig. 3) along dendrites with slightly depolarized (−64.0±0.3 mV by 16.5±3.1 pA) or hyperpolarized (−77.6±0.3 mV by −13.4±3.0 pA) states (Fig. 2a). Depolarization in this range did not induce sustained calcium influx, as indicated by the lack of a significant difference in the baseline green fluorescence (Fluo-5F; −0.8±0.6%, *P*=0.17, *n*=54, *t*-test).

Somatic hyperpolarization had bidirectional effects on bAP-evoked dendritic calcium signals, which depended on the distance from the soma (Fig. 2b,c; Supplementary Fig. 4a). In the proximal region, bAPs evoked at hyperpolarized somatic membrane potentials resulted in larger average calcium signals (20–75 μm from the soma, 12.5±1.5%, *P*=2.5 × 10^−9^, *n*=31 locations, *t*-test) compared with bAPs evoked at depolarized membrane potentials. However, the enhancement of bAP-evoked calcium signals was restricted to the proximal dendrites. In the distal dendritic region, somatic hyperpolarization had the opposite effect: the bAP-evoked calcium signals were smaller during somatic hyperpolarization (beyond 120 μm, −15.6±3.9%, *P*=0.0022, *n*=12, *t*-test). The location dependence could be clearly demonstrated by the linear correlation among individual data points (*R*^2^=0.604, *P*=2.9 × 10^−12^). Similar membrane potential- and location-dependent bAP-evoked calcium signalling was identified at physiological temperatures (Supplementary Fig. 4a). Importantly, in addition to reflecting the somatic membrane potential in a graded manner (Supplementary Fig. 5), dendritic calcium signals were also modulated by a physiological hyperpolarization elicited by G protein-coupled inwardly-rectifying potassium (GIRK) channels through mGlu2 receptor activation in the proximal dendrites^31^ (Supplementary Fig. 6).

Altogether, physiological analogue information about the somatic membrane potential location-dependently and bidirectionally modulated the bAP-evoked dendritic calcium signals in GCs. Therefore, dendritic calcium signalling is capable of extracting analogue information from hybrid bAP signals. Yet, the moderate influence of the analogue content on calcium signals enables the hybrid bAP signal to maintain its integrity and major function of transmitting the digital content, which, thus, fulfills an important criterion for analogue modulation.

### Smaller distal bAPs lead to reduced calcium influx

What mechanisms provide the functional link between the analogue state of the GCs and the bAP-evoked dendritic calcium signals? Changes in the AP waveform may affect local calcium signalling in various cellular compartments through multiple potential mechanisms. First, more hyperpolarized voltages during the repolarization phase (when majority of calcium channels are open) can promote larger peak calcium influx because of the larger driving force^32^. Second, wider (depolarized) spikes may be more effective in opening calcium channels, as has been demonstrated for narrow axonal and dendritic APs, resulting in more calcium entry^11^, ^33^, ^34^, ^35^, ^36^. Third, the smaller absolute peak amplitude of the bAPs may activate fewer calcium channels.

**Figure 3:**
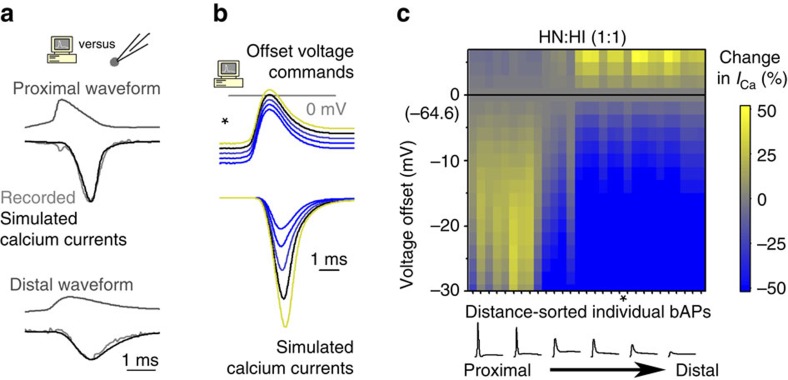
The lower peak of the distal bAPs during hyperpolarization leads to smaller calcium influx. (**a**) Comparison of simulated composite calcium currents (HN:HI, for details see Supplementary Fig. 7 and Supplementary Table 1) with recorded HVA calcium currents (in the presence of NNC55-0396) evoked by proximal and distal bAP waveforms as voltage commands. (**b**) Simulated calcium influxes after shifting the entire voltage command traces in 2 mV steps in a representative experiment (originally recorded 116 μm from the soma). (**c**) The heat map summarizes the changes of the simulated calcium influx upon offsetting the command voltage in 2 mV steps. Each column corresponds to one previously recorded bAP (six examples are shown below), which were sorted along the *x* axis according to the location of their recording. Zero voltage on the colour plot corresponds to the original recorded potential (depolarized, membrane potential: −64.6±0.6 mV). Notice the sensitivity of the small distal bAPs to small voltage shifts.

Before we directly addressed the underlying mechanisms of the effect of bAP waveforms, we established a model configuration that provided testable hypotheses based on the available calcium currents in GCs. For this aim, we recorded native calcium currents in nucleated patches (Supplementary Fig. 7a). The total calcium current (Cd^2+^-sensitive) consisted of low-voltage-activated (LVA, sensitive to T-type channel blocker, NNC55-0396 (ref. 37)) and high-voltage-activated (HVA) components. In this part of the study, we focused only on HVA components because LVA calcium currents were likely to be arbitrary overrepresented in this recording configuration^38^, ^39^, ^40^ (see below for experimental supports for the LVA exclusion). Next, we used available HVA kinetics from GCs^41^ to construct current models, which included a conventional, non-inactivating HVA calcium current (HN, based on the properties of an N-type channel) and an inactivating HVA conductance (HI, R-type). We conducted three simulation sets using these two currents individually and as a composite current (HN:HI), which consisted of both HVAs in a one-to-one ratio (maximal conductance). Among these models, the HN:HI current gave the best match with the nucleated patch HVA recordings (Fig. 3a; Supplementary Fig. 7b,c; Supplementary Table 1), but we used all three scenario to test hypotheses below.

We initially focused on the distal decrease of the bAP-evoked calcium signals by somatic hyperpolarization. Because there was a substantial reduction in the peak voltage of the bAPs in this region, we tested whether the smaller peaks were sufficient to explain the smaller calcium signals during somatic hyperpolarization. We used bAPs recorded from different dendritic regions at depolarized membrane potentials (Fig. 1) as a voltage template to simulate calcium currents. These voltage commands were subsequently shifted by 2 mV steps from +6 to −30 mV relative to the original recordings (Fig. 3b,c; Supplementary Fig. 7d). The simulations revealed that proximal bAPs did not result in smaller calcium influx, even with large negative offsets, which suggests that large proximal bAPs activate the majority of the available HVA calcium currents in GC dendrites. In contrast, the distal bAP-evoked calcium influxes were highly sensitive to small changes in the peak potentials. These findings suggest that proximal bAP waveforms achieve almost maximal HVA calcium channel activation, whereas the activation of HVA calcium currents during the small bAPs is submaximal. We confirmed this prediction by calcium current recordings (Supplementary Fig. 8). Thus, the loss of the peak of distal bAPs results in less calcium influx^20^, ^26^ during general somatic hyperpolarization (which is consistent with the local voltage modulations in pyramidal cell dendrites^16^, ^17^, ^18^), whereas, the large proximal bAPs are not sensitive to tens of mV changes in the peak amplitude.

### Faster bAP repolarization promotes increased calcium influx

**Figure 4:**
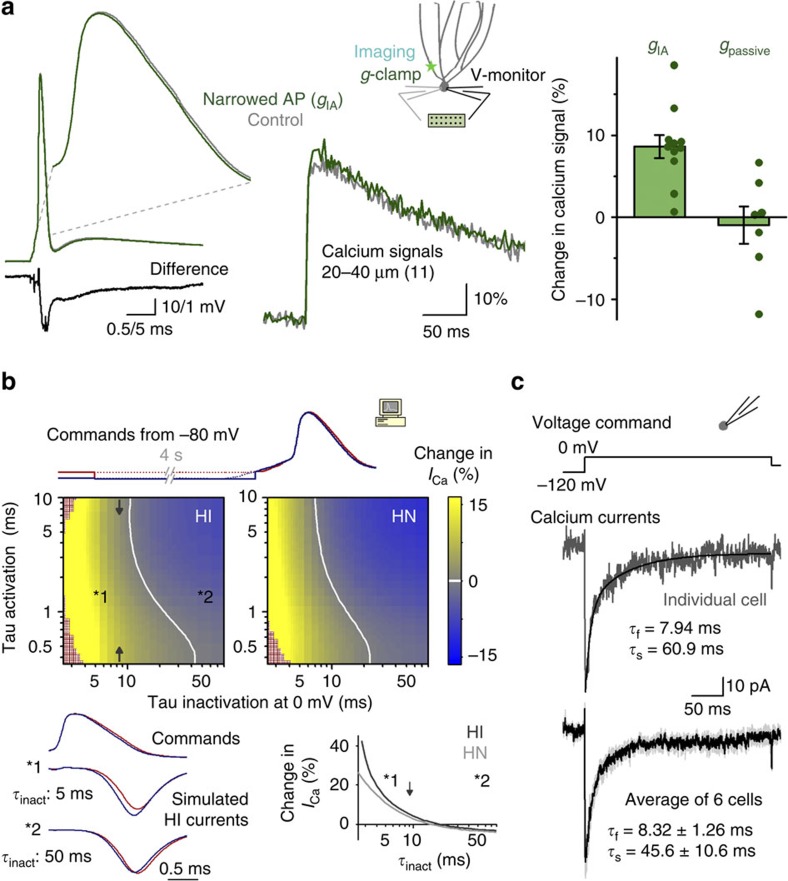
Faster repolarization promotes larger proximal dendritic calcium signals. (**a**) The acceleration of the repolarization was mimicked in conductance clamp configuration using an *I*_A_-like conductance (Supplementary Fig. 9) without affecting the AP peak and membrane potential. Traces show APs with (green, average of 11 cells) and without (grey) the *I*_A_-like conductance and their voltage difference (black). The APs with accelerated repolarization resulted in larger proximal dendritic calcium signals, unlike the APs affected by a passive conductance. (**b**) Exploration of the necessary activation and inactivation time constants that allows for membrane potential-dependent calcium influx using originally R- (HI) and N-type (HN) calcium current parameters. For these calcium current simulations, we employed an AP waveform pairs (24 μm), which were recorded at two membrane potentials (Fig. 1). These voltage commands were modified by offsetting the preceding membrane potentials to −80 mV (traces at the top) to measure the contribution of the bAP shape changes to differential calcium influx in isolation. Thus, the colour maps show the differences in the calcium influx evoked by APs with faster and slower repolarization, which derived from APs from hyperpolarized and depolarized membrane potentials. The necessity of fast inactivation time constant is demonstrated in the two example trace pairs below. The graph below is the horizontal cross-sections of the colour graphs showing the calcium influx differences at the standard activation kinetics (tau at 0 mV: 1.137 and 0.825 ms). Arrows indicate the registered inactivation time constant. (**c**) Ensemble calcium currents in GCs contain fast-inactivating components. Traces of calcium currents together with the fast (*τ*_f_) and slow (*τ*_s_) components of double exponential fits are shown from a single nucleated patch (upper trace with black fit, *R*^2^=0.916) and the average (light grey area is the s.e.m., individually fitted data, relative weights of *τ*_f_ and *τ*_s_ were 52.8±8.9% and 47.2±8.9%, respectively).

Next, we wanted to understand the mechanisms of the more surprising aspect of the analogue modulation of bAP-evoked calcium signals: their proximal enhancement during hyperpolarization. The initial clue was the difference in the proximal bAP waveforms, which repolarized faster when evoked from hyperpolarized membrane potentials. To determine the isolated effects of the faster repolarization on calcium influx, we generated an experimental model that reproduced the observed membrane potential-dependent changes in the proximal bAP waveform without other variables (for example, preceding membrane potential). Using conductance clamp, we took advantage of a high-threshold-activated transient potassium conductance (*g*_IA_), which was an ideal native tool for modulating the repolarization phase of APs (Supplementary Fig. 9). GCs were recorded with two somatic pipettes, including one pipette for monitoring the local voltage and one pipette for injecting currents, which were rapidly calculated to selectively interfere with the AP repolarization by mimicking the *g*_IA_ (Fig. 4a). The membrane potential was not affected by the mock conductance (with *g*_IA_: −68.7±0.6 mV, without *g*_IA_: −68.8±0.5 mV, *P*=0.633, *n*=11, paired *t*-test); therefore, the availability of various voltage-dependent channels were similar. Furthermore, the artificial *g*_IA_ had minimal effects on the AP peaks (with: +42.4±2.9 mV, without: +40.6±3.1 mV, *P*=0.031). The transient voltage difference between the non-injected and *g*_IA_-modified APs was comparable with the difference described between proximal bAPs evoked at depolarized or hyperpolarized membrane potentials (peak difference: −3.2±0.6 versus −5±0.6 mV, 0.75±0.17 versus 0.67±0.06 ms after AP peak, half duration: 1.35±0.18 versus 1.04±0.1 ms). Thus, the additional conductance enabled the mimicking of hyperpolarization-induced proximal bAP shape changes without affecting the membrane potential or bAP peak. We compared the bAP-evoked proximal calcium signals (20–40 μm) while alternating between the presence and absence of *g*_IA_. Relative to the controls, the APs with artificially faster repolarization evoked larger calcium signals (8.6±1.4%, *P*=0.00012, *n*=11, *t*-test). However, when a similar amount of passive conductance with a similar reversal potential was supplied by the conductance clamp circuit as a control, the calcium signals were unaffected (−0.9±2.3%, *P*=0.685, *n*=7, *t*-test). These findings indicate that artificially faster repolarization alone promotes increased calcium influx in proximal GC dendrites. Importantly, the membrane potential-dependent calcium signalling persisted during the partial blockade of AP relevant potassium currents via intracellular cesium ions (Supplementary Fig. 10), which suggests that potassium conductance (one of them used above as a tool to mimic the AP shape changes) is not necessary for the detection of the analogue content of bAPs.

### Fast inactivation is required for increased calcium influx

Next, we explored the calcium current gating requirements sufficient for faster bAP repolarization-induced larger calcium influx. We simulated calcium influx using modified waveforms of previously recorded proximal bAPs. The voltage recordings were modified to have the same preceding membrane potentials (−80 mV) until the rise of the bAPs to exclude differences in channel availabilities. Thus, in practice, the actual bAP waveforms differed only after the peak (Fig. 4b), allowing us to investigate the contribution of the activation and inactivation kinetics of the calcium currents. These simulations revealed that faster (hyperpolarized) bAP waveforms evoke increased calcium influx when HVA models had AP relevant fast inactivation. This effect was relatively independent of other current parameters, such as activation kinetics and channel type. Notably, N-type currents also became sensitive to the repolarization speed in the same way when they were supplemented with voltage-dependent inactivation. Thus, these simulations suggest that inactivation of HVA calcium currents is sufficient for the enhancement of calcium influx through faster repolarization.

To test whether the GCs' HVA channels have AP relevant fast inactivation^42^, we recorded currents in nucleated patches with long voltage steps (300 ms). The decay of isolated HVA currents (in the presence of sodium-, potassium- and T-type calcium channel blockers) were fitted with a double exponential (*τ*_fast_: 8.3±1.3 ms, *τ*_slow_: 46±11 ms, *n*=6 recordings; Fig. 4c), indicating that a substantial fraction (52.8±8.9%) of the calcium currents in the GCs adhere to the inactivation criterion predicted by the above simulations.

Next, using the HN:HI model, we tested whether the calcium current inactivation was not only sufficient but also necessary for the enhancement of calcium influx induced by faster repolarization. In the commands, the voltage before the bAPs was set to −60 mV for both the originally depolarized and originally hyperpolarized traces. Thus, as before, only the actual bAP waveforms were different, and the depolarized preceding membrane potential made the majority of inactivating HVAs unavailable. In these simulations, bAPs with faster repolarization were no longer able to evoke larger calcium influx (Supplementary Fig. 11). Similarly, in nucleated patches, the same modified commands with −60 mV preceding voltage occluded the enhancing effect of the faster bAP repolarization (−4.2±1.4%, *n*=8 recordings). Thus, when only the difference in bAP shape was included, inactivation of the HVA calcium currents was both sufficient and necessary for the enhancement of calcium influx by the faster bAP waveform. However, the waveform was not independent of other parameters, such as the preceding membrane potential, which we addressed as described below.

### Inactivating HVAs are essential for hybrid bAP signalling

**Figure 5:**
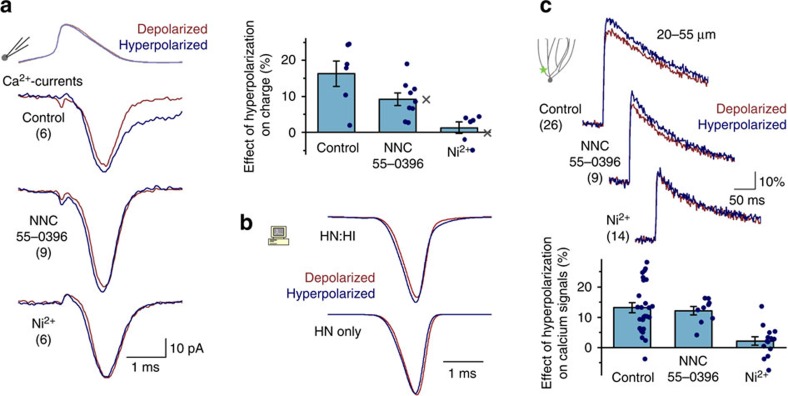
Hyperpolarization-induced enhancement of calcium influx requires inactivating HVA calcium currents. (**a**) Average traces and changes in charge (0.35–1.35 ms after the peak of bAP) of the calcium currents in nucleated patches evoked by previously recorded proximal bAP pairs (24 μm) at two membrane potentials, in control conditions and in the presence of Cav3 blocker, NNC55-0396 (10 μM), or in the presence of Cav2.3 and Cav3 blocker, Ni^2+^ (500 μM). Crosses indicate the changes predicted by simulations (see **b**). (**b**) Simulated HVA calcium currents with inactivating and non-inactivating HVA or only non-inactivating HVA components corresponding to pharmacologically isolated currents in NNC55-0396 or Ni^2+^, respectively. (**c**) Average calcium imaging traces at two membrane potentials and the effect of somatic hyperpolarization in control conditions and during the blockade of Cav3 (10 μM NNC55-0396), or Cav2.3 and Cav3 channels (50 μM Ni^2+^, the lower concentration was needed to avoid interference with imaging). Numbers indicate numbers of experiments.

The results described above predicted that fast-inactivating HVA calcium currents are sufficient and necessary for the hyperpolarization-induced enhancement of proximal bAP-evoked calcium signals. A likely candidate that meets these criteria is the R-type calcium current (primarily mediated by Ca_V_2.3, CACNA1E channels), which is present in GCs, it is activated at high voltages and inactivated at low voltages^43^, ^44^. To experimentally dissect the contribution of different classes of calcium channels, we selectively inhibited specific subsets of calcium currents in nucleated patch recordings and during calcium imaging and compared the analogue modulation of the calcium influx. In these calcium current recordings (Fig. 5a), the voltage commands were the originally recorded bAP traces and included all components of the changes of the proximal bAPs (for example, the different membrane potential and repolarization). In the control conditions (without calcium channel blockade), the hyperpolarized bAP waveform evoked larger calcium currents; the most prominent change was produced in the T-type component (which was likely to be overrepresented in nucleated patches^38^, ^39^, ^40^). When T-type channels were selectively blocked with NNC55-0396, the hyperpolarized bAP waveforms remained effective in eliciting larger calcium influx (9.2±1.7%, *P*=0.00073, *n*=9, *t*-test), confirming that calcium influx can be enhanced by the involvement of HVA channels only. The recordings with NNC55-0396 correspond to the HN:HI simulation scenarios (Fig. 5b). In contrast, when R-type currents were removed in addition to T-types by 500 μM NiCl_2_ (corresponds to the HN only simulations), the calcium influx evoked by the hyperpolarized proximal bAP waveforms was no longer increased compared with the calcium influx evoked by the depolarized bAPs (1.3±1.6%, *P*=0.44, *n*=6, *t*-test). These findings confirm that the inactivating HVA R-type currents are indeed sufficient and necessary for the larger calcium influx observed during hyperpolarized states.

Next, we investigated the contribution of various calcium channels in an even more intact situation—where only a specific subset of calcium currents is blocked and the AP propagation, native morphology and other channel functions were present—by imaging calcium signals in the proximal dendritic regions of GCs at two membrane potentials. Similar to the nucleated patch recordings, selective elimination of T-type currents by NNC55-0396 did not affect the modulation of bAP-evoked proximal calcium signals by the somatic membrane potential (Fig. 5c, control: 13.1±1.6%, *P*=2 × 10^−8^, *n*=26; NNC55-0396: 12.1±1.3%, *P*=2 × 10^−5^, *n*=9, *t*-test). However, in the presence of the R- and T-type blocker, NiCl_2_ (50 μM), the enhancing effect of hyperpolarization on local calcium influx was greatly reduced (2.1±1.4%, *n*=14; analysis of variance (ANOVA): *P*=6 × 10^−5^, Bonferroni Ni^2+^ versus NNC55-0396: *P*=0.0046). Note that the SNX-482 toxin, which specifically inhibits R-type channels among different calcium channels, acts also on A-type potassium currents^45^; thus, it cannot be used for calcium channel identification using this experimental arrangement. Altogether, multiple lines of experimental evidence confirm the predictions of our simulations that the fast-activating, fast-inactivating HVA R-type channels are necessary and sufficient for the hyperpolarization-induced enhancement of proximal bAP-evoked calcium influx.

### Theta-dependent dendritic calcium signalling by hybrid bAPs

**Figure 6:**
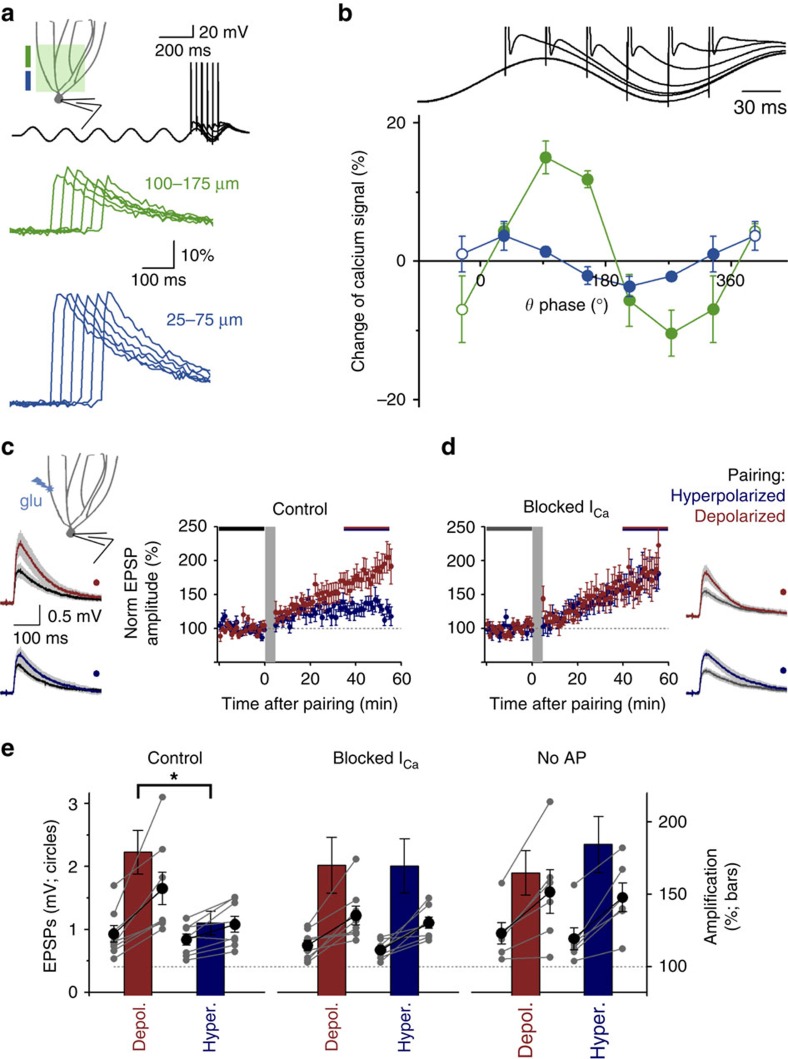
Physiological impacts of the analogue content of the hybrid dendritic bAPs. (**a**) Average calcium signals evoked by single APs, which were preceded by five sine waves in the theta range (5.2 Hz). (**b**) Changes of the bAP-evoked calcium signal amplitudes during the theta cycle relative to rest at proximal (light blue) and distal (green) regions. For better visualization, the first and last data points in the theta cycle are shown after and before the actual data (open symbols). See also Supplementary Fig. 4. (**c**) Average traces (*n*=8 and 8 cells) before and after pairing of glutamate uncaging-evoked distal EPSPs (150–200 μm) with postsynaptic APs (300 pairing at 1 Hz, timing ±4 ms; Supplementary Fig. 13). The pairing was made either at depolarized (red, −62.5±0.5 mV) or hyperpolarized (blue, −81.4±0.5 mV) membrane potentials. The graph shows the differential long-term changes of EPSP amplitudes depending on the pairing membrane potential (*n*=8 and 8 cells). (**d**) Same experimental arrangement as in **c** except that calcium channels were blocked with Ni^2+^, nifedipine, NNC55-0396 and ω-conotoxin (*n*=8 and 8 cells at −62.3±0.2 and −81.1±0.4 mV, respectively). (**e**) Summary of the long-term changes in control conditions, with inhibited calcium channels and using a pairing protocol that lacks postsynaptic firing (*n*=8, 8, 8, 8, 6 and 6, respectively, at hyper- and depolarized pairing protocols). Bars show the average relative effects of pairing at the two membrane potentials in the three conditions (right axis, *P*=0.013, *P*=0.99, *P*=0.45, respectively, *t*-test, averages of the 30–50 min period after pairing) and circles indicate the absolute EPSP amplitudes before and after pairing (left axis).

What are the potential functional significances of the analogue modulation of bAPs? First, we tested whether the calcium signals were capable of reflecting physiologically relevant membrane potential fluctuations, such as theta oscillations. To accomplish this aim, we employed spinning-disk confocal imaging, which allows simultaneous sampling of large dendritic regions. Single APs were preceded by 5.2 Hz oscillations (between −62.2±0.7 and −83.9±0.3 mV) evoked via current injection (Fig. 6a,b). APs during different phases of the ongoing theta cycles resulted in different calcium signals (*P*=0.0066, *n*=5 cells, one-way repeated measure ANOVA, Greenhouse–Geisser correction for non-sphericity) in the distal dendritic region (100–175 μm). Between 40° and 160°, the signals were larger than the signals evoked without voltage fluctuations at rest (−73.3±0.3 mV); however, during the trough phase (219°–339°), the signals were smaller in the distal dendritic region. In contrast, the phase dependence was less pronounced in the proximal region (25–100 μm; *P*=0.062, *n*=5 cells, one-way repeated measure ANOVA). Thus, the analogue modulation of bAPs enables theta-phase-dependent dendritic calcium signalling in the GCs, and this phenomenon also occurs at physiological temperatures (Supplementary Fig. 4b). Further investigation revealed the potential time frames, in which the two opposite effects of the hyperpolarization operated on dendritic calcium signals (Supplementary Fig. 12). Namely, the distal effect was instantaneously available, whereas the proximal enhancement required prolonged (>200 ms) hyperpolarization. The similarity of this time course to the recovery from inactivation of HVA currents in GCs (362±136 versus 422±291 ms) further supports the crucial role of the putative R-type calcium currents.

### The analogue content of hybrid bAPs modify synaptic plasticity

Finally, we investigated whether the analogue content of bAPs modulates plasticity at dendritic synapses as an attempt to find evidences for biological entities, which are potentially capable of detecting the relatively small contribution of the analogue content to the hybrid bAP signals. Given the complexity of the activity-dependent regulations of synaptic strength^4^, we evoked long-term synaptic plasticity via the involvement of only the recorded postsynaptic cell and only a few synapses (smaller than 2 μm regions) on its dendrites activated by glutamate uncaging. Single postsynaptic APs were paired with distal dendritic glutamate uncaging stimulation of the same small regions (300 pairing at 1 Hz; the two events occurred nearly synchronously, within ±4 ms, 150–200 μm). This pairing protocol led to a larger net long-term potentiation when it was performed at depolarized somatic membrane potential (179.1±15.1%; MP: −62.6±0.5 mV, *n*=8; Fig. 6c,e; Supplementary Fig. 13) compared with hyperpolarized pairing (130.2±8.1%; −81.4±0.5 mV, *n*=8, *P*=0.013, *t*-test, note that the relatively slow expression of changes is likely due to the weak induction protocols). Common voltage-dependent mechanisms may explain this observation^4^. Therefore, to directly prove their associative role, we uncoupled bAP and calcium influx by using inhibitors against all HVA calcium channels, which underlie the hyperpolarization-induced reduction of calcium influx in this region (Fig. 3), in the same experimental arrangement (Ni^2+^ against R- and T-types, ω-conotoxin GVIA against N-type, NNC55-0396 against T-type and nifedipine against L-type). In other control experiments, we also assessed the membrane potential dependence of the long-term plasticity induction without postsynaptic firing. In both conditions, the induction protocols at de- or hyperpolarized membrane potentials resulted in similar long-term plasticity (Fig. 6c,e; 170.0±19.4% versus 169.7±18.6%, *P*=0.99, *n*=8 and 8 cells; 164.7±15.4% versus 184.4±19.5%, *P*=0.447, *n*=6 and 6). Thus, these controls showed that the differences in plasticity depended only on voltage-gated calcium channels activated by bAPs and, thus, they excluded the possible involvement of other voltage-dependent mechanisms, such as the different functional availability of NMDA receptors^17^, ^46^. Even though the observed synaptic plasticity changes may not share each expression mechanisms, the necessity of bAP and bAP-associated calcium influx suggests that the mechanisms of the analogue modulation described above in this study enable synapses interpreting the analogue content of the hybrid bAPs.

## Discussion

Here we demonstrated that bAPs in the dendrites of GCs are subject to analogue modulation by somatic membrane potentials. The first important step in this phenomenon is that analogue information is distance-dependently retained by the bAP waveform. Second, the graded changes in the bAP waveforms are reflected by local dendritic calcium signalling. Thus, as hybrid signals, bAPs carry both digital timing information about spiking activity and analogue information about the overall state of the cells. Notably, our findings highlighted that hybrid dendritic signalling is available during at least two physiological conditions; therefore, hybrid dendritic signalling increases the capacity of neurons and their synapses to code, relay and decode various types of neuronal information^5^, ^13^.

The moderate extent of the analogue modulation seems to be an important feature of the hybrid signalling. First, the low relative weight allows that the analogue content cannot override the primary digital timing information Thus, each state-dependently modulated bAPs maintains its primary role to carry digital feedback signal to the dendrites about the output activity of the neurons. Although local dendritic membrane potential is capable for more effective interference with local bAP signalling and synaptic plasticity^17^, these active boosting effects (such as NMDA receptor-mediated mechanisms) are spatially restricted and do not carry similar analogue information for neighboring dendrites. Second, the contribution of the analogue content to the weight of hybrid bAPs in the dendrites is similar to the axonal hybrid signalling^6^; therefore, the moderate contribution of the analogue content seems to be a general and necessary feature of hybrid signalling. Third, the extent of the cell-autonomous analogue modulation is similar to the effect that can be achieved on bAP signals by localized activation of dendritic receptor pathways, such as the GABA_B_ (γ-aminobutyric acid) receptor-mediated downregulation of calcium channel functions^47^, ^48^, albeit the underlying mechanisms are fundamentally different. Forth, the sensitive mechanisms that we described here allow that physiological membrane potential differences are faithfully reflected by the dendritic hybrid signals, from which synapses can translate the analogue content as differential weight changes after plasticity induction. Voltage changes arise mostly from dendritic excitatory synapses, thus, the somatic membrane potential is not an independent modulator from the viewpoints of activated dendrites. However, somatic membrane potential reflects every dendritic branch and additional sources of voltage changes, such as the diverse perisomatic GABAergic inputs^49^, supramammillary glutamatergic inputs^50^ and non-synaptic sources^51^. Thus, the somatic membrane potential contains more information than the local dendritic potentials, which is continuously relayed back to each dendrite equally via the hybrid bAPs. A support for the biological relevance of the somato-dendritic signalling direction is our plasticity experiments, where synapses were activated on a single branch by uncaging, yet, the somatically introduced small, but physiological membrane potential difference was able to overcome the effects of the local voltage changes and impose a modulatory effect.

The morpho-physiological design of GC dendrites makes them ideal for hybrid bAP signalling because each dendrite independently collects inputs and the overall activity converges onto the soma^25^, ^52^. The exchange of analogue input signals between individual branches is weak as a result of the asymmetric voltage propagation. Thus, the dispersion of analogue information by bAPs is an effective way of notifying each dendrite about the ongoing overall subthreshold activity. Although GCs are well suited for hybrid dendritic signalling, the underlying mechanisms we described here are present among other cell types. Thus, analogue information presumably modulates bAP signalling in other cells as well. Indeed, similar effects of the somatic membrane potential on single spike-evoked calcium signals have been observed in medium spiny neurons of the striatum^53^.

Interestingly, the location dependence of dendritic hybrid AP-signalling matches the anatomical organization of the excitatory inputs to the GCs. Namely, the proximal third of the dendritic field (the average dendritic length in our sample was ∼300 μm) is innervated by ipsilateral and contralateral mossy cells^54^, whereas axons from the medial entorhinal cortex form synapses in the middle section of the dendrites. This input localization co-aligns with the opposing modulation of bAP-evoked calcium signals by the somatic membrane potential. These spatial profiles enable the differential regulation of active synapses depending on their origin and the analogue state of the postsynaptic cell when it fires APs. In this framework, the medial entorhinal cortical inputs will coincide with larger bAP-evoked calcium influx when the membrane potential is depolarized, whereas calcium influx is smaller at the mossy cell synapses. We provide evidences that changes in the analogue content of hybrid bAPs modulate the long-term synaptic plasticity of distal synapses, and that the contribution of the analogue modulation is fully available during ongoing somatic voltage fluctuations in the theta frequency. Therefore, medial entorhinal inputs that are preferentially active during different phases of the theta will be differentially modified by synaptic plasticity. It should be noted that our results provide only one example of the differential modulation of synaptic strength. Interference with synaptic plasticity, especially with the cooperativity rules of induction, by somatic membrane potential is not unprecedented^46^. These observations raise the possibility that hybrid signalling is available in other principal cell types. Therefore, the contribution of the analogue content carrying hybrid bAPs to these observed effects should be clarified, in addition to the well-established voltage-dependent NMDA receptor functions (which were excluded in our experiments). Moreover, the contributions of each aspects of hybrid signalling to synaptic plasticity described in this study, such as its timing, location and state- dependence, must be resolved also in future studies.

One surprising aspect of our results on the underlying mechanisms was that the slight narrowing of dendritic bAPs was not necessarily associated with smaller calcium signals. This is in contrast to axonal APs, where an increase of AP width leads to greater calcium influx in various cell types^55^, ^56^, ^57^, ^58^ (but see ref. 59), including the axons of our subject cells^34^. This proximal action was unprecedented also in the dendrites, where local voltage has been shown to modulate bAPs similarly to what we have described in the distal regions^16^, ^17^, ^18^. Notably, axonal APs are narrower than somatic or dendritic spikes, and widening of fast axonal APs can further increase the number of activated channels. In contrast to axons, in the dendrites—under our experimental conditions, in the proximal region—the activation of calcium channels is nearly complete. Therefore, widening is not capable of opening more channels. As a consequence, calcium influx is primarily determined by the deactivation and inactivation kinetics. Indeed, our results point to a critical and novel role for fast calcium current inactivation, which is sufficient and necessary for sensitivity to subtle AP shape changes and for the amplification of these changes as differences in calcium influx.

## Methods

Experimental procedures were made in accordance with the ethical guidelines of the Institute of Experimental Medicine Protection of Research Subjects Committee (22.1/1760/003/2009).

### Solutions and chemicals

Our standard artificial cerebrospinal solution contained 126 mM NaCl, 2.5 mM KCl, 26 mM NaHCO_3_, 2 mM CaCl_2_, 2 mM MgCl_2_, 1.25 mM NaH_2_PO_4_ and 10 mM glucose (equilibrated with 95% O_2_ and 5% CO_2_ gas mixture). Slices were cut in a cutting solution consisting of 85 mM NaCl, 75 mM sucrose, 2.5 mM KCl, 25 mM glucose, 1.25 mM NaH_2_PO_4_, 4 mM MgCl_2_, 0.5 mM CaCl_2_ and 24 mM NaHCO_3_. External solutions were equilibrated with 95% O_2_ and 5% CO_2_. Unless stated otherwise pipettes were filled with an internal solution containing 90 mM K-gluconate, 43.5 mM KCl, 1.8 mM NaCl, 1.7 mM MgCl_2_, 50 μM EGTA, 10 mM HEPES, 2 mM Mg-ATP, 0.4 mM Na_2_-GTP, 10 mM phosphocreatine disodium and 15-25 μM Alexa Fluor 594 (pH=7.25 adjusted with KOH). Chemicals for the intra- and extracellular solutions were purchased from Sigma-Aldrich, blockers were from Tocris or Alomone and fluorophores were from Invitrogen.

### Slice preparation and microscopy

Hippocampal slices (350 μm) were prepared from adolescent Wistar rats (P23-P36, both sexes) in ice-cold cutting solution in an orientation optimized to preserve the mossy fibre tract in the CA3 area^60^. After cutting, slices were kept at 32 °C for at least 30 min and then stored at room temperatures until the recordings. With the exception of the data shown in Supplementary Fig. 4, experiments were performed at room temperatures (23–28 °C) using an upright microscope (Eclipse FN-1; Nikon) equipped with a high-numeric aperture objective (Nikon 1.1 NA, Apo LWD 25 × W or Nikon 0.8 NA Apo 16 × W), with a sCMOS or CCD camera (Andor Zyla 5.5 controlled by NIS Elements software or Hamamatsu C7500) for IR-DIC (900 nm) video microscopy and with a confocal system (see below).

### Dual somato-dendritic recordings

To obtain dual current clamp recordings, we first established stable dendritic patch-clamp recordings with the aid of the IR-DIC optics. After a brief (no more than 10 min) dialysis of the cell, the position and morphology of fluorescently labelled parent soma was defined using the red channel of the confocal system and then patched under IR-DIC control. Recordings were terminated if substantial changes appeared in the dendritic electrical properties (that is, in the access resistance, in the input resistance or in the background noise) during the dialysis or during patching the soma. Patch pipettes were pulled from thick-wall borosilicate glass tubes (inner diameter: 0.86–0.75 mm, outer diameter: 1.5 mm, Sutter or Hilgenberg) coated with dental wax to further reduce the pipette capacitance (mean pipette capacitance was 6.85±0.12 pF, no correlation with the distance from soma, *R*^2^=−0.04, *P*=0.635, linear fit). Recordings had at least 2 GΩ seal resistance before the break-in. The average series resistance at the monitoring pipettes was 118.7±10.5 MΩ (weakly correlated with the distance from soma, *R*^2^=0.13, *P*=0.04684), which was fully compensated and constantly monitored using fast large current steps within each recorded trace. Pipette capacitance was optimally neutralized (as evidenced by similar threshold and peak values of APs at the monitoring pipettes during various holding current conditions; 0.1±0.06 pF remaining capacitance) using the built-in bridge balance and capacitance neutralization circuits of the amplifier (MultiClamp 700B, Molecular Devices). Traces were low pass filtered at 10–20 kHz and digitized at 40–250 kHz using a Digidata 1440 A interface (Molecular Devices). Currents to adjust the membrane potential and to evoke action potentials were delivered only through the somatic controlling pipette and we quantified the action potential parameters recorded only via the dendritic monitoring electrode. The results in Supplementary Fig. 1 demonstrate that the above recording conditions allows for the reliable detection of small changes in the AP shapes. Single action potentials were evoked alternately from depolarized and from hyperpolarized membrane potential (49.4±5.5 and 1.5±3.3 pA, respectively, resulting in −64.6±0.6 and −77.2±0.6 mV membrane potential) using brief current steps (2–5 ms, 0.85–2 nA). GC identity was confirmed by anatomical and electrophysiological properties: characteristic accommodating firing in response to long-lasting (1 s) depolarizing current injection, single action potentials were followed by prominent after depolarization, and polarized axonal and dendritic orientation. Note that cells in our sample showed matured GC properties^61^ in respect to their input resistance (196.2±11.7 MΩ ranging from 105.6 to 313.7 MΩ measured in current clamp mode at rest), and resting membrane potential (77.8±1.6 mV ranging from −83.3 to −68.6 mV, determined immediately after break-in), and their soma located within the GC layer. Cells were discarded from the analysis if the access resistance was larger than 200 MΩ (Supplementary Fig. 1) or if the cell had an initial resting membrane potential more depolarized than −65 mV. For quantification, traces (4–6 individual sweeps in both conditions) were time aligned to the maximal rate of the somatic AP and averaged.

### Calcium imaging with conventional scanning confocal microscopy

Cells were patched with 183 μM Fluo-5F and 15 μM Alexa Fluor 594 salt containing intracellular solution. Imaging (Nikon Eclipse C1 Plus, EZ-C1 software) started at least 30 min after break-in to allow equilibration of the dyes within the dendrites. Up to five dendritic locations were defined by the red fluorescence signal (543 nm laser, 4.8–5.9 μW power at the tip of the objective) 25–45 μm from the surface of the slice. Scan lines (10.24 μm length, 610 lines per second) were positioned over dendritic shafts. During the experiments, red channel was used to monitor the position of the line and its position was readjusted or the objective was refocused if necessary. If the baseline and the decay of the action potential-evoked green signals changed >30% of the initial values the experiments were excluded from analyses. Single action potentials were evoked using 2 ms-long 1.6 nA current injections. In those experiments where steady-state somatic membrane potential dependence of dendritic calcium signals was investigated, the APs were evoked alternately from slightly depolarized and hyperpolarized membrane potentials relatively to rest (16.5±3.1 pA and −13.4±3.0 pA, respectively, resulting in −64.0±0.3 and −77.6±0.3 mV). To avoid interference with the imaging lower NiCl_2_ concentration was used in the imaging experiments than in the nucleated patches (50 μM versus 500 μM). The 50 μM NiCl_2_ blocks majority of T-type channels and a large fraction of R-type channels (half-maximal inhibitory concentration for R-type channel block ∼20–50 μM (refs 44, 62, 63)). The mGlu2 agonist, DCG IV (Supplementary Fig. 6), was applied by two different approaches either by pressure application (5–8 μM) from a small tapered glass capillary near to the imaging sites or by bath application (1 μM). The first approach allowed the comparison of alternately recorded calcium signals multiple times at the same locations during control condition and when mGlu2s were activated. Whereas the bath application allowed the concurrent monitoring of the DCG IV-induced changes at multiple dendritic sites and maintaining a constant agonist concentration. In the latter approach, control calcium signal measurements were conducted on equal number of traces from the prior baseline and subsequent washout periods. The results from the two approaches were similar, thus the data were merged. The background corrected fluorescence of the Fluo-5F channel (Δ*F*/*F*_0_, 488 nm, 5 μW) was analysed from 0.64 to 1.92 μm regions (2–6 pixels) by integrating the area of the green signal in a 15 ms-long time window following the action potential after subtraction of the baseline. Average calcium signals are shown without filtering, whereas traces from single experiment have been smoothed (5:1) for illustration purposes (Fig. 2a). After the calcium imaging, the morphology of the dendrites and the distance of the imaging sites from the soma were retrieved in three-dimensional by using the red channel at higher intensities then during the imaging sessions.

### Calcium imaging with spinning-disk confocal microscopy

To image large areas of the dendritic field simultaneously, we employed spinning-disc confocal imaging (Andor Revolution XDv system including Yokogawa CSU-X1 Nipkow disc, Andor iXon 860 EMCCD Camera and Andor iQ2 software) using the same labelling and recording protocols, as above either at room temperatures (23–26 °C) or at near physiological temperatures (35–36 °C, Supplementary Fig. 4). Images were captured at 93.75 frames per second and 20 frames per second rates consecutively for the green (488 nm laser excitation, 125–230 μW) and red channels (561 nm, 32–58 μW). Fluorescence was quantified as the changes in the peak green signal (average of 6 or 7 frames after APs minus a 13–16 frame-long baseline period) relative to the steady red signal except for (Supplementary Fig. 5), where raw green fluorescence measurements (that is, no normalization to red signal and no baseline adjustment) are shown for detecting any potential change in baseline calcium levels. Theta oscillation was evoked by sinusoid current injection with 48±6 pA in both polarities. Single APs were preceded by at least five full theta cycles (5.2 Hz) and each imaging trace was separated by at least 25 s.

### Calcium current measurements in nucleated patches

Somatic membranes were isolated together with the nucleus by slowly retracting the pipette under gentle negative pressure. The internal solution was composed of :133.5 mM CsCl, 1.8 mM NaCl, 1.7 mM MgCl_2_, 1 mM EGTA, 10 mM HEPES, 2 mM Mg-ATP, 0.4 mM Na_2_-GTP and 10 mM phosphocreatine disodium. The extracellular solution contained elevated calcium concentration (5 mM), 1 μM TTX and 5 mM 4-AP. All voltage protocol started with a 4 s-long baseline to allow calcium channels to reach their steady-state availability at the given baseline potential. Previously recorded AP waveforms were applied as voltage-clamp command to assess AP relevant macroscopic calcium currents. For constraining the model (Supplementary Fig. 7), we applied a 3 ms-long test pulse to 0 mV from −80 mV, and previously recorded bAP traces as voltage command. For the measurement of the inactivation of the native calcium currents (Fig. 4c), 300 ms-long test pulses (0 mV) were applied from −120 mV to maximize the contribution of inactivating calcium channels. For testing the completeness of the calcium current activation (Supplementary Fig. 8), two previously recorded bAP waveform pairs have been used as a template voltage command and these were extended with various lengths (0.75–5 ms) of steady voltage before they reached their peak. Thus, the repolarization phase, when the majority of bAP-activated calcium currents flow, remained as in the native waveform. Sweeps were collected with 12 or 25 s intervals and digitized at 50 or 80 kHz. Leak and capacitive current components were subtracted using P/−4 method. We noticed significant rundown of calcium currents during longer recordings. Therefore, our analysis restricted to those traces that were recorded in the first 12 min after establishing the whole-cell configuration. The activation and inactivation of the currents were fitted with monoexponential and with biexponential functions, respectively. The bAP-evoked currents (Figs 3, 4, 5) were quantified as the baseline subtracted area of a 1 ms-long time window starting 0.35 ms after the peak of the AP command.

### Computational modelling

We performed single compartment computer simulations using the NEURON^64^ simulation environment (version 7.2, downloaded from http://neuron.yale.edu). Based on the assumption that the recorded macroscopic HVA calcium currents can be described by the combination of non-inactivating and inactivating components (Fig. 3), we constructed two model currents using Hodgkin–Huxley formalisms. Activation kinetics were constructed on the basis of previously published channel properties recorded from GCs^41^. Namely, N-type conductance served as template to construct a fast, non-inactivating (HN) HVA model, whereas we used the R-type current for the HVA, inactivating (HI) model. Equations describing the voltage-dependent activation and inactivation of the HI current were:


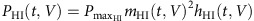



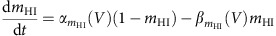



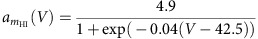



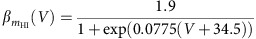



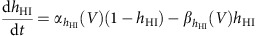



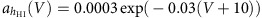



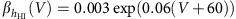


Equations describing the HN current were:


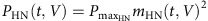



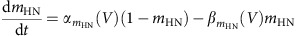



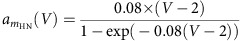



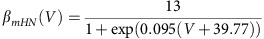


Calcium current was calculated as follows:


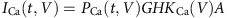


where *P*_Ca_ was the membrane permeability for calcium ion, *A* was an amplitude factor and *GHK*_Ca_ was a modified constant field equation^65^, ^66^ in the form of:


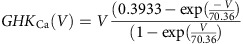


where V was the modelled membrane potential. The net calcium permeability was the linear combination of individual model current activation:


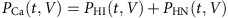


The total theoretically maximum permeability was fixed to 1 in all simulation conditions (HI only, HN only and HN:HI) to keep *P*_Ca_ in the range of 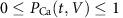. The activation time constants were calculated using the formulation of:


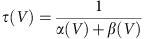


Model was incorporated onto a single compartment with three segments (area=100 μm^2^, *c*_m=_1 μF cm^−2^, *R*_a_=35.4 Ωcm), where the membrane potential was forced to follow previously recorded AP waveforms or voltage steps. The elicited calcium currents were analysed in the middle of the compartment. Integration time steps were 2.5 or 12.5 μs for those experiments, which validated the models (Supplementary Fig. 7) and 0.5 μs for those simulations, where we tested the effects of AP shape differences on (Figs 3b, 4b and 5b; Supplementary Fig. 11). Effects on the model calcium currents were quantified in the same way as described for the calcium current recordings except in case of simulations on Fig. 4b, where we measured current integrals in a larger time window (5 ms) to avoid potential variances introduced by the highly diverse time courses of the currents. We excluded those simulations, which resulted in unrealistic currents due to unlikely kinetics (that is, extremely fast inactivation and extremely slow activation rates. For details see Supplementary Fig. 14).

### Two electrode dynamic-clamp experiments

We employed a software based dynamic-clamp system to manipulate the AP repolarization phase. Current injections were calculated by the StdpC2012 software^67^ (downloaded from http://sourceforge.net/projects/stdpc) through a MIO-16E-4 analog/digital card (National Instruments Inc.) based on voltage signals of the measuring electrode and an A-type current. Using a Hodgkin–Huxley-type formalism for a native A-type inactivating potassium conductance (4–10 nS maximal conductance) at a clamp rate of 83 kHz or higher, we were able to interfere with action potentials. For the mock conductance, 4-AP-sensitive (5 mM) outward currents were isolated in nucleated patches (1 μM TTX, *n*=6). Conductance values were calculated assuming ohmic channel behaviour and a potassium equilibrium potential of −90 mV. Time constants of activation and inactivation were measured by fitting the rising phase (from 0.25 ms after onset to the peak) and the decay phase (in a 100 ms-long time window after the peak) with monoexponential functions (Supplementary Fig. 9). The clamp current of A-type potassium conductance model was generated as follows:


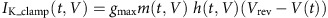


where *I*_K_clamp_ was the calculated model current, *g*_max_ was the maximal conductance, *m* and *h* were gating variables, *V*_rev_ was the reversal potential of the model conductance and *V* was the measured membrane potential of the cell. Gating variables were modelled using the formalism of:


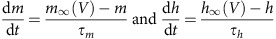


where *m*_∞_ and *h*_∞_ were the steady-state values, and *τ*_*m*_ and *τ*_*h*_ were voltage-dependent time constants modelled as:


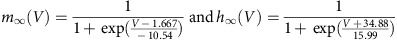



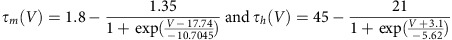


For passive conductance measurements, we used the same Hodgkin–Huxley model, but the activation was instantaneous, whereas inactivation was extremely slow (10^9^ ms); therefore, conductance was always fully activated. Reversal potential of the model current was set to −70 mV to minimize the current flow at rest. Calcium imaging on dual-patched conductance clamped cells (membrane potential: −68.7±0.6 mV) was performed as described above.

### Spike timing-dependent plasticity protocol

To evoke plasticity at well-defined synaptic locations, we employed glutamate uncaging and the evoked responses were paired with individual APs. During the experiments, cells were patched using IR-DIC imaging and intracellularly filled with Alexa Fluor 594 for at least 15 min. Next, an intact and complete dendritic region was selected within 30–50 μm from the surface of the slice. During the long course of these experiments (at least 90 min), the imaging site was monitored and adjusted if necessary. Glutamate-EPSPs were evoked by 405 nm laser illumination^31^, ^68^ (0.74 ms-long pulses repeated two to three times at 1 kHz) at a small dendritic spot (<2 μm) 150–200 μm from the soma (mean distance: 179.8±2.2 μm, *n*=44 cells) using the conventional confocal system described above. A concentration of 1 mM MNI-glutamate was supplied in the recirculated recording solution (10 ml). Before and after the pairing, EPSPs were tested every 20 s at −71.4±0.1 mV. After a 20 min-long baseline period, the pairing protocol was applied, which consisted of 300 AP-EPSP pairings at 1 Hz (timing range: ±4 ms, mean: −0.82±0.4 ms). During the pairing, the EPSPs were evoked by the same conditions as during the control and test periods except the stimulation frequency and membrane potential (depolarized: −62.2±0.3 mV or hyperpolarized: −81.1±0.3 mV). High resistance (>15 MΩ) recording pipettes were used to keep the cells intracellular milieu intact, and, thus, to preserve the capability for synaptic plasticity. Data acquisition were stopped if the cell experienced lower access resistance than 50 MΩ. Access and input resistances were monitored in each trace by injecting a −10 pA 500 ms-long and a −200 pA 0.3 ms-long pulses. Calcium channel blockers were bath applied in the presence of 1 mg ml^−1^ bovine serum albumin. After the recordings, the morphology of the dendrites and the distance of the uncaging site from the soma were retrieved in three-dimensional using the confocal system in the red channel. EPSP amplitudes were quantified in a 5 ms-long window at the peak. Traces with spontaneous events were excluded from analysis. To test whether the plasticity was dependent on the membrane potential during the induction, the changes of the amplitude of the EPSPs were compared (that is, depolarized versus hyperpolarized induction) using Student's *t*-test.

### Data analysis and statistics

Data were analysed using Molecular Devices pClamp, OriginLab Origin, Microsoft Excel and Stimfit^69^. Voltage values are presented without correction for the liquid junction potential. Somatic distances were measured on confocal z stack images taken after the end of experiments using the Alexa Fluor 594 signal. Differences of the values from one or two different groups were tested using parametric, paired or unpaired, two-tailed Student's *t*-tests (abbreviated as *t*-test in the text). Normality of the data was analysed with Shapiro–Wilks test. Data are presented as mean±s.e.m. In case of the experiments on membrane potential effect in the presence of T-type channel blocker (Fig. 5c) Grubb's test identified a significant outlier (*P*=0.00027). Thus, this value (48.6%) was excluded from the further analysis. The removal modified the average from 15.8±3.8% to 12.1±1.3%, which does not change the conclusions of the experiment. The same analysis did not identify outliers in the control (*P*=0.953) and in the Ni^2+^ (*P*=0.19) data sets.

### Data availability

The authors declare that all other relevant data supporting the findings of this study are available on request and from szabadicslab.koki.hu.

## Additional information

**How to cite this article:** Brunner, J. and Szabadics, J. Analogue modulation of back-propagating action potentials enables dendritic hybrid signalling. *Nat. Commun.* **7**, 13033 doi: 10.1038/ncomms13033 (2016).

## Supplementary Material

Supplementary Information Supplementary Figures 1-14, Supplementary Table 1 and Supplementary References

Peer Review file
