# Supplementary material for: Analogue modulation of back-propagating action potentials enables dendritic hybrid signalling
Source: Nat Commun. 2016 Oct 5;7:13033. doi: 10.1038/ncomms13033 (PMC5059477; doi:10.1038/ncomms13033)
Supplement: Supplementary Information — Supplementary Figures 1-14, Supplementary Table 1 and Supplementary References [file ncomms13033-s1.pdf]

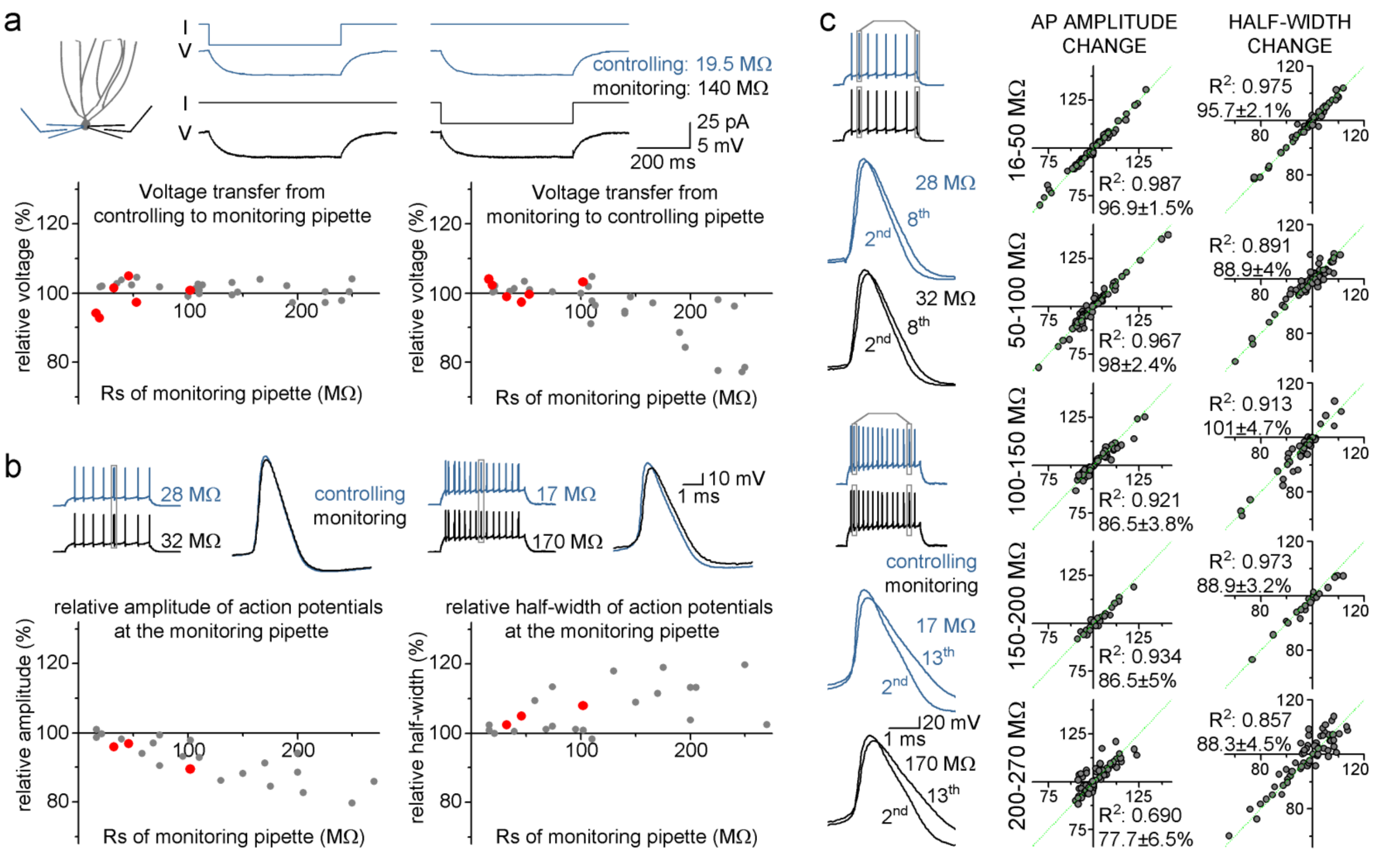

Supplementary Figure 1. Effects of high access resistance (Rs) on the recorded AP parameters.

Rationale: The small diameter GC dendrites can be whole-cell patched only with small pipettes. Smaller pipettes result in higher Rs. We implemented several controls in the experimental design in order to obtain reliable recordings in spite of the high Rs. During the recordings of the data for **Fig. 1** in the main text, inclusion of slow (500-600 ms, 5-20 pA) and fast test pulses (0.5 ms, 200 pA) in every traces on both pipettes allowed for the continuous monitoring and immediate full compensation of the changes of Rs. Furthermore, to obtain comparable results independent of the size of the recorded structure similarly small pipettes were used for all dendritic and somatic voltage monitoring. Finally, in the main experiments we always used relative values between similar recording conditions. Namely, AP parameters were analyzed from intermingled recordings, in which only the membrane potentials were different. In this supplementary data set, to appreciate the reliability of the high Rs recordings for our experimental questions, GCs were recorded with two somatic pipettes. As in the case of somato-dendritic recordings in the main data, one controlling pipette with relatively low Rs was used for injecting currents and the other pipette was for monitoring the voltage, whose Rs ranged from 18-250 MΩ. Note that the voltage data from the controlling pipettes was not analyzed in the main text because of the temporal distortion of the voltage signal caused by the current injection.

(**a**) The propagation of steady-state voltage signals from the small Rs controlling pipette was reliably followed in the monitoring pipette (left panel), whereas, the voltage signal is distorted when the higher Rs pipettes are used for current injections (right panel). Therefore, high access pipettes were not used for current injection (hence the name, monitoring pipette). Each dot represents individual cells. The data points indicated by red dots derived from those recordings (i.e. same traces), from which data was collected for **Fig. 1**. (**b**) Comparison of the peak amplitudes and half-widths of the same APs at the low Rs controlling pipettes and monitoring pipettes with various Rs values in dual somatic recordings. (**c**) Comparison of the changes of the same APs detected at the monitoring and controlling pipettes during long trains. This analysis exploited the known broadening and amplitude reduction of the GC APs during long trains. Thus, this approach allowed for the comparison of sensitivity of recordings to subtle changes of AP shapes, which were similar to those changes that have been the subject of our study. The obtained data were grouped according to the Rs of the monitoring pipettes. Two representative recordings are shown on the left. On the graphs, each dot represents the change of individual APs relative to a reference AP in the same traces at the controlling pipettes (x-axis) and at the monitoring pipettes (y-axis). The reference AP was chosen from the middle portion of the trains. Adjusted R^2 is shown for each graph together with slope of the linear fits on the data. This analysis indicates that up to 200 MΩ the majority of the values fell close to the ideal (100% slope); thus, our recording conditions were sensitive enough to detect the small changes of the bAP shapes.

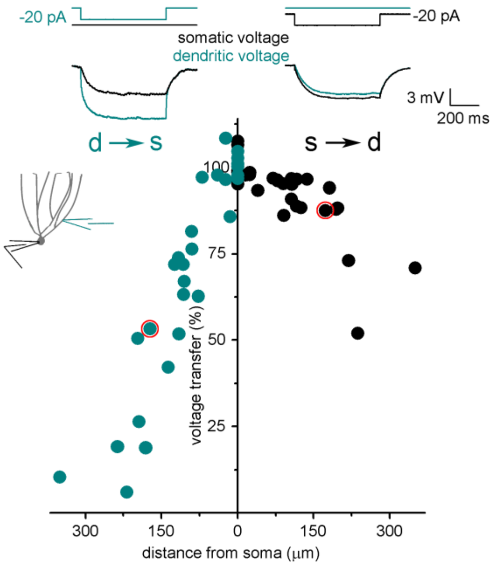

Supplementary Figure 2. Asymmetric propagation of steady-state voltages from soma to dendrites and from dendrites to soma in GCs.

Rationale: We replicated previous observations concerning asymmetric voltage propagation from soma to dendrites and dendrites to soma in GCs[1,2]. The morphological and biophysical properties of GC dendrites enable more effective voltage propagation in the soma to dendrite direction and this was an important prerequisite for the effective modulation of dendritic processes by somatic membrane potential changes.

Traces on top are average somatic (black) and dendritic (cyan, recorded at 173 µm from the soma) voltage responses evoked by local dendritic (left) or somatic (right) current injection (-20 pA, 600 ms). Graph shows the relative attenuation of steady-state voltage responses of individual recording pairs (n=28 dual recordings). Red circles indicate the example recording.

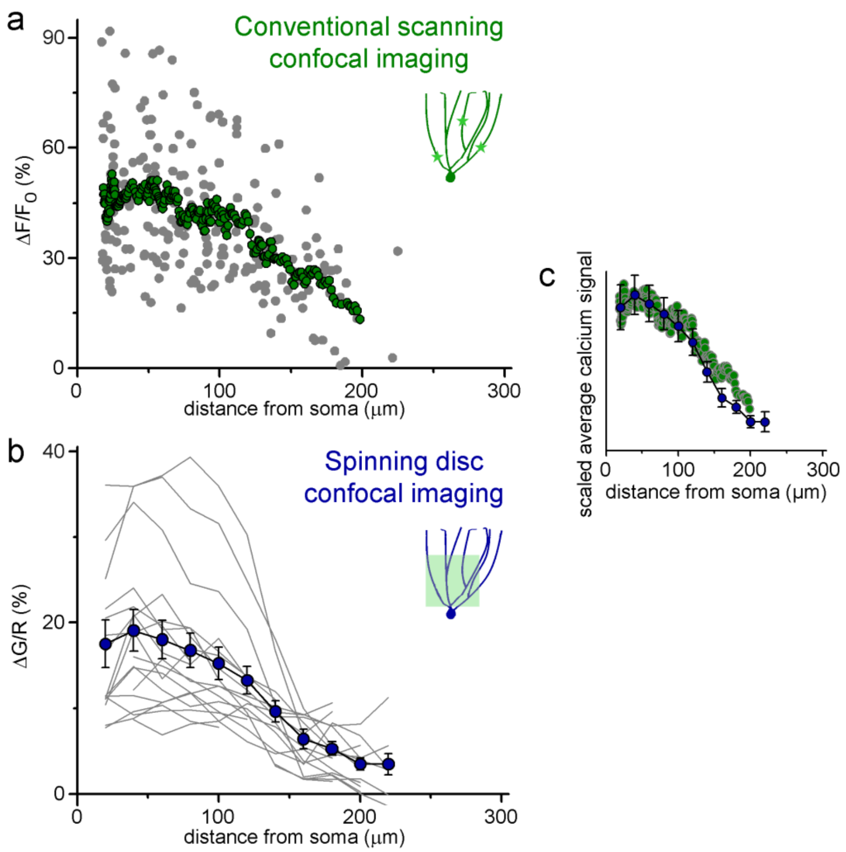

Supplementary Figure 3. Location-dependency of the bAP-evoked dendritic calcium signals in GCs measured by scanning confocal microscopy and spinning disc confocal imaging.

Rationale: Conventional scanning confocal imaging allows for the spatially precise measurements of individual dendritic spots, whereas, the spinning disc method provides better throughput and simultaneous imaging of large dendritic region. Here we tested whether the results of the two approaches give comparable results compared to each other and compared to previous findings[1].

(**a**) Conventional scanning confocal imaging. Gray symbols show the individual peak fluorescence values ($\Delta F/F_0$, signal from 183 µM intracellular Fluo-5F dye) measured at different distances from soma (n = 189 locations) in individual cells. Green symbols show the running average of 13 distance-sorted individual points. (**b**) Spinning disc confocal imaging. Gray lines show the ΔG/R measurements (change of Fluo-5F signals divided by the signals from morphological dye, Alexa Fluor 594) along the dendrites of individual cells (partitioned to 20µm-long segments) and the symbols show the average results. (**c**) The y-axis was made dimensionless by arbitrary scaling for the visual comparison of the distance-dependence of the calcium signals obtained by the two imaging methods.

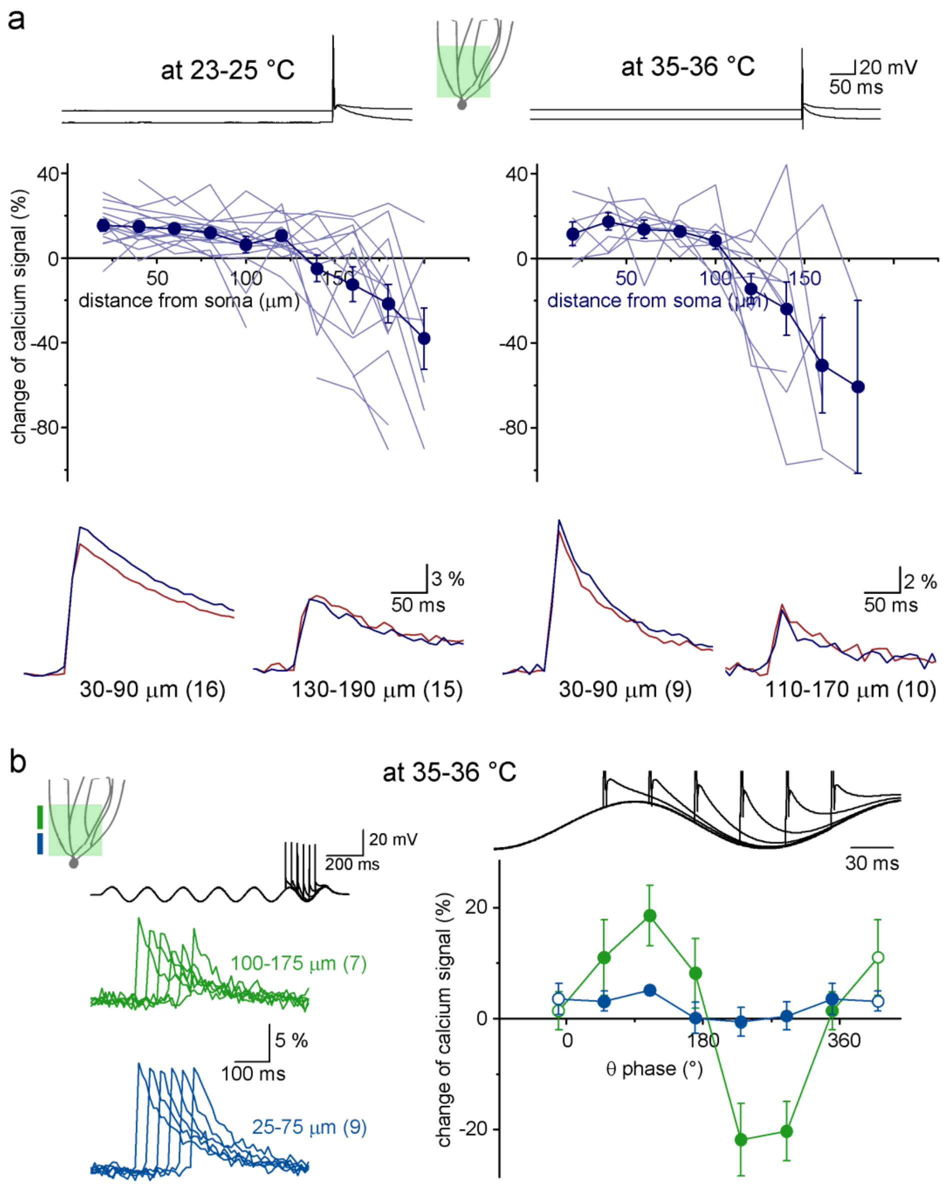

Supplementary Figure 4. Location-dependent bidirectional effects of somatic membrane potential on bAP-evoked dendritic calcium signals at room- and at near physiological temperatures.

Rationale: Experiments in the main text were conducted at room temperatures. For validating the physiological availability and relevance of hybrid dendritic signaling it was important to repeat the key observations at physiological temperatures.

(**a**) Light blue lines mark the effects of hyperpolarization on local calcium signals along the dendritic distance in individual experiments and dark blue symbols show the average results at 23-25°C (left, see

the similar spatial profile in **Fig. 2**) and at physiological (35-36°C, right) recording temperatures measured by spinning disk confocal imaging. Calcium signals were averaged in 20 µm-long dendritic segments. The average ΔG/R calcium signal traces from the proximal and distal data at two membrane potentials (blue, hyperpolarized, -79.5 ± 0.1 and -78.5 ± 0.3 mV; red, depolarized, -62.8 ± 0.1 and -64.1± 0.3 mV) are shown below. The numbers of imaging experiments included in the averages are indicated in parenthesis. (**b**) APs were evoked during subthreshold membrane potential oscillation in the theta range (5.2 Hz, at 35-36°C). The APs were preceded by five sine waves. The graph on the right shows the changes of the bAP-evoked calcium signal amplitudes during the theta cycle relative to rest at proximal (light blue) and distal (green) regions. APs during different phases of the ongoing theta cycle resulted in different calcium signals in the distal dendritic region (100-175 µm; $p=8.6x10^{-4}$, n=7 cells, One-Way Repeated Measure ANOVA, Greenhouse-Geisser correction for non-sphericity). Whereas, the calcium signals in proximal region did not depend significantly on the phase of the theta (25-75 µm; p=0.421, n=8 cells). For better visualization the first and last data points in the theta cycle were repeated (open symbols) after and before the actual data. Similar phase-dependence has been observed at room temperatures (**Fig. 6**).

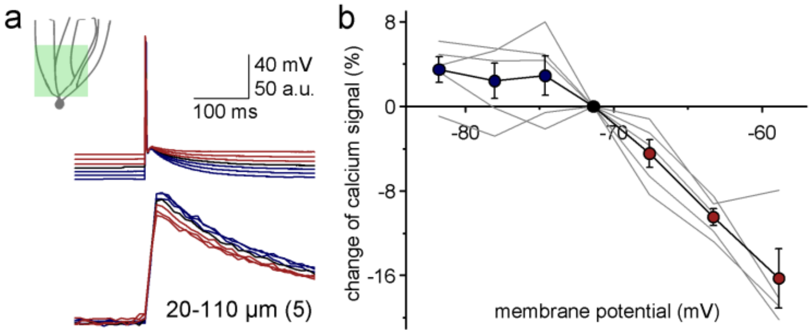

Supplementary Figure 5. Graded relationship between the membrane potential and the amplitudes of the bAP-evoked calcium signals.

Rationale: For matching the definition, it was important to show that calcium signals follow the membrane potential in analog manner within physiologically relevant voltage range.

(**a**) Action potentials and the related calcium signals in the proximal dendritic region (average of 5 cells, 20-110 µm from the soma) were evoked from resting (black, -71.4 ± 0.2 mV) or from slightly hyper- (blue; -74.6 ± 0.3, -78 ± 0.3 and -81.8 ± 0.3 mV, respectively) or depolarized (red; -67.6 ± 0.2, -63.3 ± 0.3 and -58.9 ± 0.3 mV, respectively) membrane potentials. Notice the lack of change in the baseline calcium signal (raw traces are shown in arbitrary units) in these membrane potential ranges, which suggests that calcium currents were not activated significantly at these membrane potentials and that the differences of the imaged calcium signals were not due to changes of the baseline signal. (**b**) Quantification of the membrane potential dependence of bAP-evoked calcium signals. The signal amplitudes were normalized relative to calcium signal amplitudes evoked at resting membrane potential (black symbol) in each cells (gray curves show data from the individual cells).

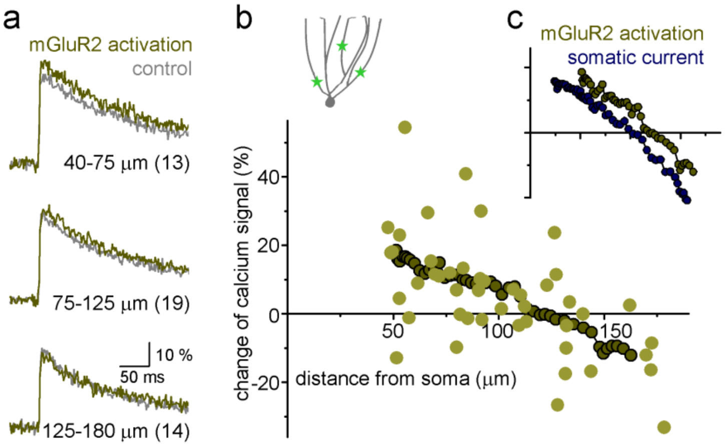

Supplementary Figure 6. Analog modulation of dendritic calcium signals by GIRK-mediated proximal dendritic hyperpolarization.

Rationale: Throughout the manuscript we employed somatic current injection to evoke membrane potential changes. To test whether more physiological effectors have similar effects on bAP-evoked calcium signals as somatic current injections we employed the mGluR2 activation. Dendritic mGluR2 receptors are localized in the proximal dendritic region of GCs and evoke GIRK-channel-mediated hyperpolarization[3]. First, to exclude the possibility that mGluR2 and its G-protein cascade directly modulate dendritic calcium signaling in GCs in addition to activating this potassium conductance, we tested the effect of the mGluR2 agonist DCG IV in the presence of the GIRK channel blocker tertiapin-Q (0.5 μM). In these experiments, in which the hyperpolarization was specifically eliminated (somatic resting membrane potential in the presence of tertiapin-Q only: -72.9 ± 0.6 mV, and in tertiapin-Q and DCG IV: -73.3 ± 0.6 mV), mGluR2 activation was not able to elicit changes in the measured calcium signals (45-90 μm: -1.6 ± 6.2%, p=0.8, n=9 locations; 97-160 μm: -0.7 ± 6.1%, p=0.9, n=9) indicating that any effect of mGluR2 activation on calcium influx could be attributed to the GIRK-mediated hyperpolarization. As with somatic hyperpolarization, mGluR2 activation by DCG IV application alone (which hyperpolarized the somatic resting membrane potential from -71.0 ± 0.6 mV to -74.9 ± 0.5 mV) bi-directionally modulated the dendritic bAP-evoked calcium signals.

(**a**) Average calcium imaging trace-pairs along the dendrites in control conditions (gray; n=46 locations from n=20 cells) and when proximal dendritic mGluR2 receptors were activated by the agonist, DCG IV (brown). As in **Fig. 2**, here we employed the spatially precise conventional confocal imaging. (**b**) The graph on the right shows the individual data points (light brown) and the running average (brown) of the distance dependent effects of mGluR2 activation on local calcium signals. (**c**) The similar analysis for the effect of somatic hyperpolarization was shown in blue for comparison (see **Fig. 2c**).

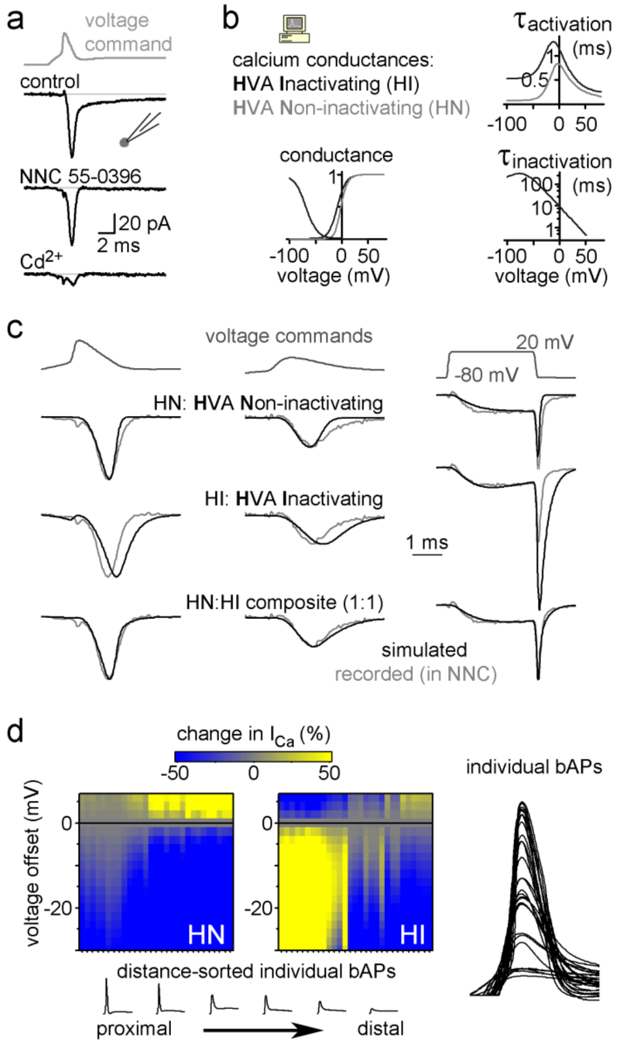

Supplementary Figure 7. Simulated calcium currents in GCs.

Rationale: We employed a model configuration that provided testable hypotheses based on the available calcium currents in GCs. For this, we isolated native calcium currents in nucleated patches from GCs and constrained HVA current models from GCs[4] to the measured parameters. We used two current models, which included a conventional, non-inactivating HVA calcium current (HN, based on the properties of an N-type channel) and an inactivating HVA conductance (HI, R-type). We conducted three simulation sets using these two currents individually and as a composite current (HN:HI composite), which consisted of both HVAs in a one-to-one ratio (maximal conductance). Note that LVA (T-type) calcium currents were likely to be arbitrary overrepresented in nucleated recording configuration (e.g. refs[5-7]).

(**a**) Isolated calcium currents evoked by AP-waveform in nucleated patches (NNC55-0396 blocks Ca_V3, T-type channels; Cd^{2+} blocks all voltage dependent calcium channels). (**b**) Parameters of two simulated calcium currents (light gray: HN, dark gray: HI). (**c**) Comparison of individually simulated and their composite (HN:HI) calcium currents with recorded HVA calcium currents (in the presence of NNC55-0396) evoked by three different voltage commands. The amount of simulated conductance was constrained to match the peak amplitudes (AP) or steady-state amplitudes (square pulse) of the measured calcium currents. Among the tested, the composite current (consisting of equal maximal conductance of the HI and HN currents) gave the best approximation of the recordings (see also **Supplementary Table 1**). (**d**) Same simulations as in **Fig. 3** using HI and HN calcium currents alone. The heat maps summarize the changes of the simulated calcium influx upon offsetting the command voltage in 2 mV steps. Each column corresponds to one previously recorded bAP, which were sorted along the x-axis according to the location of their recording (six example commands are shown below the graphs). Zero voltage on the color plot corresponds to the original recorded potential (depolarized, membrane potential: -64.6 ± 0.6 mV). Every AP commands are shown on the right.

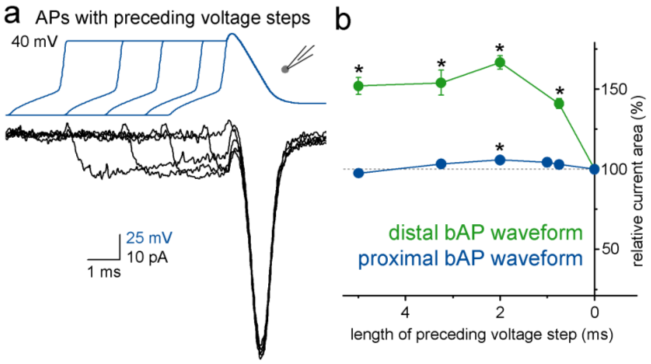

Supplementary Figure 8. Nearly complete activation of calcium channels by proximal bAP waveforms.

Rationale: For the mechanisms underlying the proximal enhancement by hyperpolarization-induced bAP narrowing it was crucial that these bAPs maximally activate the available channel population. To test how close is the calcium channel opening to the maximal by the different bAPs we artificially extended them with constant voltage steps (at +40 mV for proximal bAPs and at 0 mV for distal bAPs). If the bAP waveforms do not activate nearly all calcium channels, extending the bAP voltage command was expected to increase the calcium influx. Note that currents were measured at the descending phases of the modified bAP waveforms (tail currents); thus, the driving force was identical in each protocol.

(**a**) The extension the proximal bAP waveform (recorded at 24 µm) with steady voltage at their peaks (+40 mV for proximal bAP) did not increase significantly the amount of activated calcium currents in nucleated patches indicating that the bAP waveform alone already activated the majority of the available channels. Traces show the average of all raw currents (i.e. without normalization, n=12 patches, recorded in the presence of NNC55-0396, TTX and 4-AP) elicited by a bAP voltage command extended by 5, 3.75, 2, 0.75 or 0 ms. (**b**) The graph shows the area of modified proximal (light blue, n = 12 patches) and distal (green, n = 9 patches) bAP-evoked calcium currents relative to that of native bAP (0 ms) in the same nucleated patches. In contrast to the proximal bAP waveform, extension the distal bAP waveform (0 mV, collected at 106 µm) did increase calcium currents indicating that smaller bAPs activate only a fraction of the available calcium channels and, consequently, small changes in their peak amplitude can effectively change the calcium influx. Asterisks mark significant differences (t-tests).

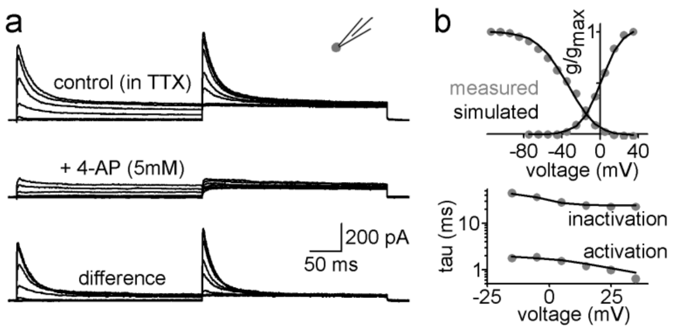

Supplementary Figure 9. Native potassium currents in GCs, which has been employed in the dynamic clamp experiments to narrow APs.

Rationale: The experiment on **Fig. 4a** was designed to investigate the isolated contributions of repolarization changes to the membrane potential-dependence of bAP-evoked calcium signals. Thus, the influences of AP-shape can be investigated in isolation from the actual membrane potential changes, which can alter other factors such as the differential availability of voltage-gated channels. For this aim, we looked for a tool, which can be employed in dynamic clamp experiments to mimic the narrowing of APs. We characterized the parameters of an inactivating potassium current in GCs, which were used as a template for the conductance fed by the computer in the conductance clamp experiments. Note that presence of these currents did not demonstrate that they were responsible for the membrane potential dependent changes of the bAP shape (see also **Supplementary Fig. 10**). We have employed them only to mimic the bAP shape changes.

(**a**) Outward currents (average of 6 experiments) recorded from nucleated patches in control conditions (top) and in the presence of 5 mM 4-AP (middle) to isolate inactivating potassium component (difference current, bottom). Currents were elicited using 300 ms long test pulses to +55 mV preceded by a series of conditioning pulses (300 ms, from -105 mV to 65 mV, 10 mV increment). Only odd traces are shown for clarity. (**b**) Peak conductance and activation and inactivation time constants of the recorded inactivating currents (gray symbols) along with the derived clamp conductance parameters (black curves).

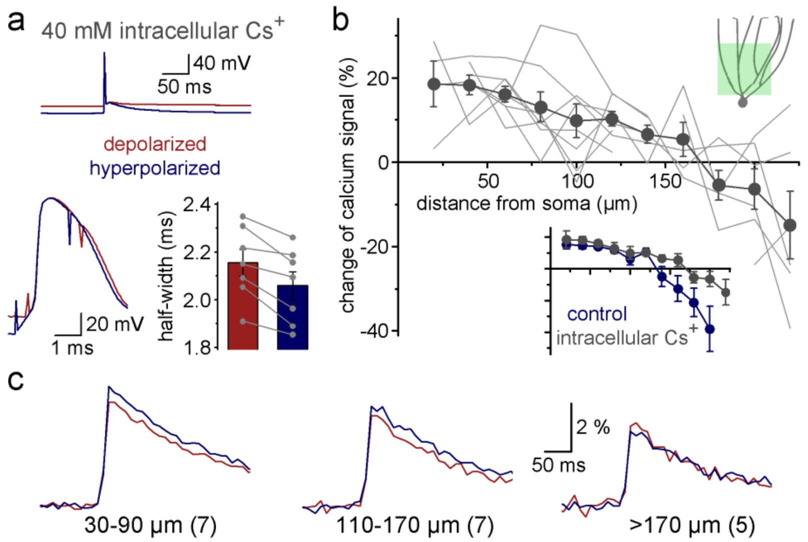

Supplementary Figure 10. Analog modulation of bAP-evoked dendritic calcium signals by membrane potential was maintained during partial blockade of potassium currents.

Rationale: Blocking a significant fraction of potassium channels was expected to diminish the proximal enhancement of calcium signals during hyperpolarized membrane potentials if potassium channels were directly involved in the membrane potential-dependent shaping. On the other hand, in the distal region the hyperpolarization-induced reduction of the calcium signals was expected to be shifted toward the end of the dendrites due to the more efficient back-propagation of the APs into the dendrites during potassium current blockade. The more efficient propagation is expected to be promoted both by the higher membrane impedance and/or broadened bAPs.

(**a**) Average membrane potential traces during spinning disk imaging while the cells (n=7) were recorded with intracellular 40 mM CsCl (replacing KCl). The expanded view of an AP-pair from an example cell shows the widened APs by Cs^+ and the persisting membrane potential dependent repolarization speed. 10 µM NNC55-0396 was added to the recording solutions to prevent T-type current activation. The graph summarizes the half width of the APs at hyperpolarized (2.06 ± 0.06 ms) and depolarized (2.16 ± 0.06 ms, p=0.003, n=7, paired t-test) membrane potentials. (**b**) Light gray lines show the effects of hyperpolarization on local calcium signals along the dendrites in individual experiments (n=7 cells) and dark gray symbols show the average results in the presence of 40 mM intracellular Cs^+. The control profile of the hyperpolarization-induced changes of local calcium signals (blue) is shown for comparison in the inset. Calcium signals were averaged in 20 µm-long segments. (**c**) Average ΔG/R calcium signal traces from three different dendritic regions at two membrane potentials (blue, hyperpolarized; red, depolarized, numbers in parenthesis indicate the number of experiments included in the average).

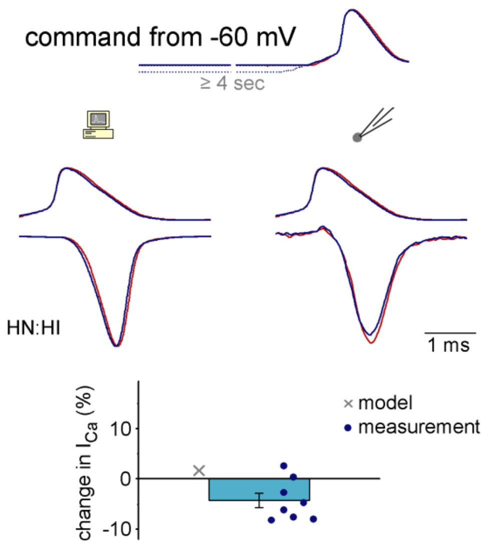

Supplementary Figure 11. Inactivation of HVA currents by physiological steady-state depolarization eliminates the narrow AP-induced calcium influx enhancement.

Rationale: The simulations predicted (**Fig. 4b**) a crucial role for the inactivation of the HVA channels. Here we tested whether low (i.e. physiological) voltage is effective to elicit this inactivation. For this aim we investigated calcium influx in a similar scenario as in **Fig. 4b** except the preceding membrane potentials, which were set to -60 mV for both the depolarized and hyperpolarized voltage commands. Thus, only the AP shape remained different between the two commands. We predicted that if the inactivation of HVA currents play important roles, in contrast to the observation with -80 mV preceding membrane potentials, the narrower AP shapes should not result in larger calcium influx when the different AP waveforms were preceded with physiological depolarizations. We addressed this question both with HI:HN simulations and with nucleated patch recordings of native currents.

The upper trace shows the modified voltage commands (blue: originally hyperpolarized, red: originally depolarized) with a 4 sec long preceding step at -60 mV. Currents were simulated by standard activation and inactivation kinetics. And calcium current were elicited in standard nucleated patch recording conditions (n=8 cells) by the same voltage commands. The lower graph shows the summary data (bar), individual recordings (blue symbols) and the simulated results (X) of the differences between calcium influx elicited by the "hyperpolarized" waveform relative to the currents during "depolarized" waveform.

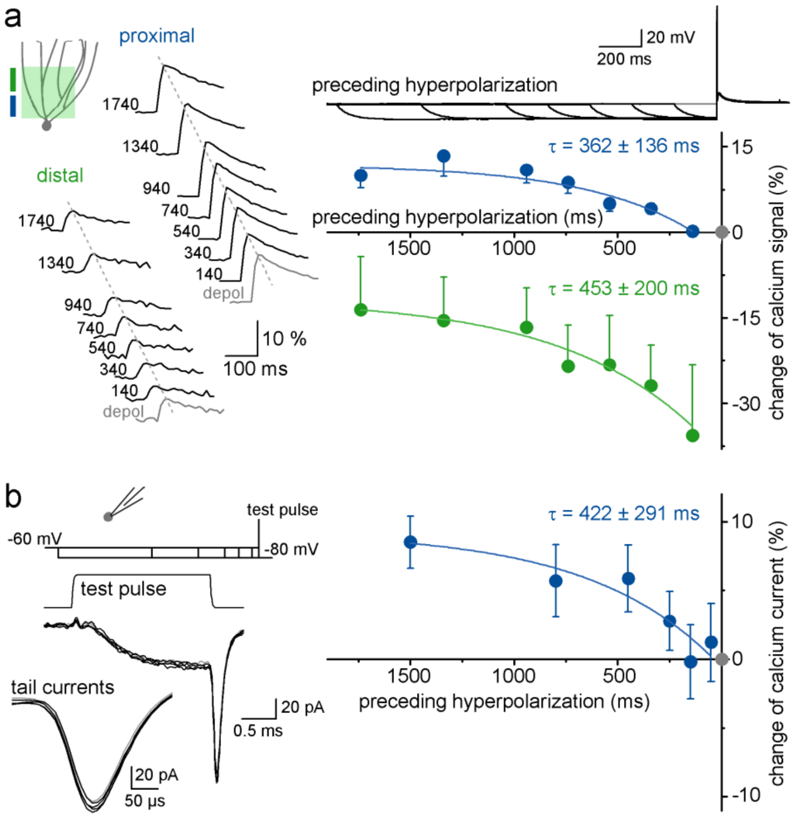

Supplementary Figure 12. The time course of the development of the effect of hyperpolarization on bAP-evoked calcium signals.

Rationale: The development of the effect of somatic hyperpolarization on dendritic calcium signals is important for determining the temporal domains, in which hybrid signaling is available (see **Fig. 6**). Because of the proposed important role of the inactivation of the HVA currents in the membrane potential-dependent calcium influx we hypothesized similar time courses for the dependence of calcium signal enhancement on the length of the hyperpolarization and for the recovery time course from inactivation of the HVA currents. Here we measured and compared these two parameters.

(**a**) Calcium signals in the proximal (light blue) and distal (green) dendrites evoked by APs preceded by somatic hyperpolarization with various length (-79.8 ± 0.7 mV; 140 - 1740 ms) or elicited from depolarized membrane potential (-62.5 ± 0.2 mV, gray traces). The traces show the average of all experiments and the length of the preceding hyperpolarization was indicated next to them. Dash lines indicate the peak of those calcium signals, which were evoked from depolarized membrane potentials. On the right, calcium signal amplitudes are shown relative to the depolarized values (0 ms; gray symbol) together with single exponential fits (proximal R^2=0.915, n=15 cells; distal R^2: 0.896, n=11, 4 cells did not have sufficient amount of distal dendrites in the imaged area). (**b**) HVA calcium currents (in the presence

of NNC55-0396) evoked by square pulses (at +20 mV, 2 ms; test) preceded by voltage steps to -80 mV for various periods (50-1500 ms). Average currents (n=17 patches) are shown with 150, 450, 800 and 1500 ms long preceding -80 mV steps and with -60 mV preceding voltage (gray). On the right, the normalized tail current areas are plotted against the length of the preceding voltage steps to -80 mV (relative to depolarized voltage, 0ms, gray) together with single exponential fit (R^2=0.837).

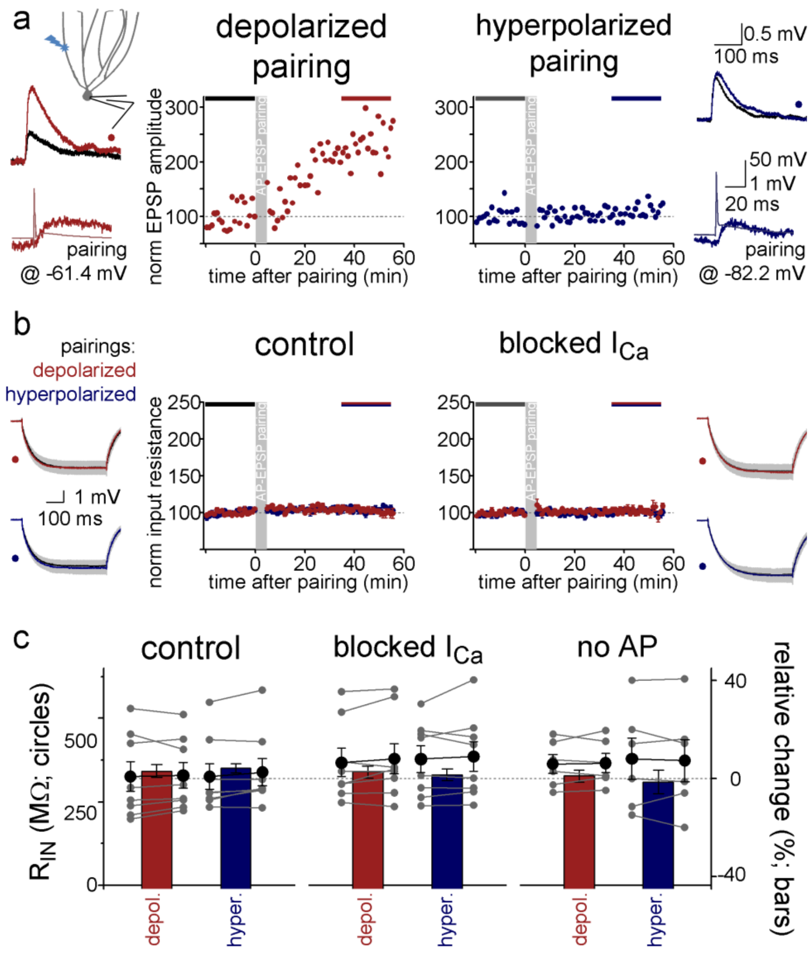

Supplementary Figure 13. Stable intrinsic postsynaptic excitability following plasticity induction.

Rationale: During the investigation of the plasticity of glutamate-uncaging EPSPs it was important to monitor the stability of the postsynaptic input resistance for excluding the possibility of intrinsic excitability changes. Here we also show two individual examples with traces for the somatic membrane potential-dependence of the induction of synaptic plasticity.

(**a**) Two individual experiments showing the plasticity of glutamate-uncaging EPSPs paired with individual postsynaptic APs, which were evoked either from depolarized (left panels in red) or from hyperpolarized (right in blue) membrane potentials. The upper traces show the glutamate-uncaging EPSPs before (black) and after pairing (red or blue). Below are the actual pairing protocols (300 pairing at 1 Hz, within 4 ms, average of all 300 pairings, **Fig. 6c-e**) the relative timing of EPSPs and APs in these two experiments. The graphs summarize the EPSP amplitudes before and after the pairing protocol (indicated by gray area) in the two example experiments. Each dot represents the average of 3 evoked events. (**b**) Stability of the input resistance of postsynaptic cells before and after pairing glutamate-uncaging EPSPs with postsynaptic firing at two membrane potentials in control conditions (left) and in the presence of

blockers for somato-dendritic calcium channel (right). Current injection steps were included in every trace. The traces show the average of all experiments before (black) and after (blue or red) pairing and the gray area is the s.e.m. of the experiments. The graph shows the Rin during the experiments. The values were normalized to the baseline period (before pairing) in each experiment. (**c**) Summary of input resistance before and after pairing in the six different conditions. The connected dots indicate raw input resistance data from individual experiments before and after pairing, whereas the bars show the average normalized input resistances after the pairing (p=0.284, p=0.066, p=0.299, p=0.539, p=0.697, p=0.782, respectively, t-test, n = 8, 8, 8, 8, 6 and 6).

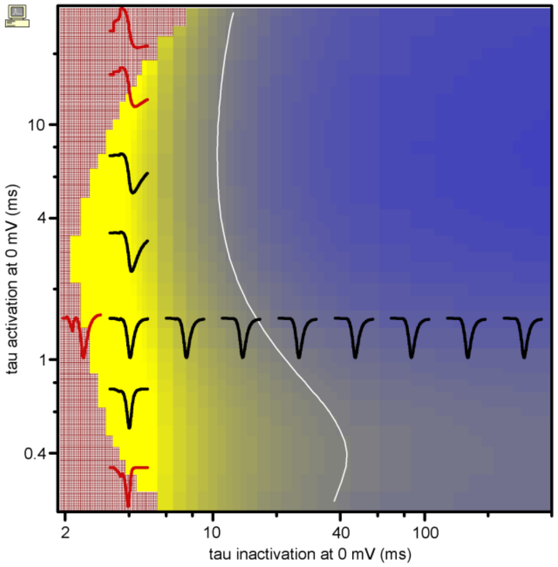

Supplementary Figure 14. Normalized HI calcium current waveforms superimposed onto the graph from **Fig. 4b** to show the position of modelled currents in the parameter space. Black traces were included to the analysis whereas red traces show currents profiles which failed to satisfy our criteria for realistic calcium currents. Specifically, traces were excluded if a double peak current rose with a first peak as large as the one-third of the second peak or too small currents were evoked (i.e. the peak current was five times smaller than the baseline).

milliseconds	**I_{Ca} in NNC**	**HN-type**	**HI-type**	**HN:HI(1:1)**
**somatic AP waveform**	(n=6)			
peak-to-peak time (from AP peak to I_{Ca} peak)	0.93 ± 0.01	1.02	1.26	1.04
width at half maximum	0.77 ± 0.01	0.63	0.96	0.66
20%-80% rise time	0.41 ± 0.02	0.44	0.56	0.45
decay tau (80%+1ms)	0.26 ± 0.01	0.15	0.43	0.25
**dendritic AP waveform**	(n=6)			
peak-to-peak time (from AP peak to I_{Ca} peak)	0.72 ± 0.03	0.6	1.08	0.78
width at half maximum	1.03 ± 0.06	0.92	1.59	1.38
20%-80% rise time	0.51 ± 0.05	0.42	0.68	0.63
decay tau (80%+1ms)	0.59 ± 0.06	0.3	0.66	0.95
**square pulse from -80 mV to +20 mV**	(n=6)			
rise tau (0.3-1.3 ms after onset)	0.41 ± 0.05	0.58	0.4	0.45
decay tau (80%+5ms)	0.13 ± 0.01	0.04	0.28	0.18
tail current ratio (steady-state/peak)(%)	575 ± 77	377	896	453

Supplementary Table 1 Parameters of measured and modeled calcium currents evoked by different voltage clamp protocols

Supplementary References

1. Krueppel, R., Remy, S. & Beck, H. Dendritic integration in hippocampal dentate granule cells. *Neuron* **71**, 512-528 (2011).
2. Schmidt-Hieber, C., Jonas, P. & Bischofberger, J. Subthreshold dendritic signal processing and coincidence detection in dentate gyrus granule cells. *J Neurosci* **27**, 8430-8441 (2007).
3. Brunner, J. et al. Selective Silencing of Individual Dendritic Branches by an mGlu2-Activated Potassium Conductance in Dentate Gyrus Granule Cells. *J Neurosci* **33**, 7285-7298 (2013).
4. Li, L., Bischofberger, J. & Jonas, P. Differential gating and recruitment of P/Q-, N-, and R-type Ca2+ channels in hippocampal mossy fiber boutons. *J Neurosci* **27**, 13420-13429 (2007).
5. Doerner, D., Pitler, T.A. & Alger, B.E. Protein kinase C activators block specific calcium and potassium current components in isolated hippocampal neurons. *J Neurosci* **8**, 4069-4078 (1988).
6. Jung, H.-y., Staff, N.P. & Spruston, N. Action potential bursting in subicular pyramidal neurons is driven by a calcium tail current. *J Neurosci* **21**, 3312-3321 (2001).
7. Mouginot, D., Bossu, J.-L. & Gähwiler, B.H. Low-threshold Ca2+ currents in dendritic recordings from Purkinje cells in rat cerebellar slice cultures. *J Neurosci* **17**, 160-170 (1997).
