# Supplementary material for: Analogue modulation of back-propagating action potentials enables dendritic hybrid signalling
Source: Nat Commun. 2016 Oct 5;7:13033. doi: 10.1038/ncomms13033 (PMC5059477; doi:10.1038/ncomms13033)
Supplement: Peer Review file [file ncomms13033-s2.pdf]

Transferred manuscripts:

Editorial Note: this manuscript has been previously reviewed at another journal that is not operating a transparent peer review scheme. There are no reviewer comments or rebuttal letters for versions considered at Nature Communications.
